# Comparative Proteomic Profiling of Divergent Phenotypes for Water Holding Capacity across the *Post Mortem* Ageing Period in Porcine Muscle Exudate

**DOI:** 10.1371/journal.pone.0150605

**Published:** 2016-03-07

**Authors:** Alessio Di Luca, Ruth M. Hamill, Anne Maria Mullen, Nikolai Slavov, Giuliano Elia

**Affiliations:** 1 Teagasc, Food Research Centre, Ashtown, Dublin 15, Ireland; 2 Department of Bioengineering, Northeastern University, Boston, MA 02115, United States of America; 3 Mass Spectrometry Resource, UCD Conway Institute of Biomolecular and Biomedical Research, Belfield, Dublin 4, Ireland; University of Bologna, ITALY

## Abstract

Two dimensional Difference Gel Electrophoresis (2-D DIGE) and mass spectrometry were applied to investigate the changes in metabolic proteins that occur over a seven day (day 1, 3 and 7) *post mortem* ageing period in porcine centrifugal exudate from divergent meat quality phenotypes. The objectives of the research were to enhance our understanding of the phenotype (water holding capacity) and search for biomarkers of this economically significant pork quality attribute. Major changes in protein abundance across nine phenotype-by-time conditions were observed. Proteomic patterns were dominated by *post mortem* ageing timepoint. Using a machine learning algorithm (l1-regularized logistic regression), a model was derived with the ability to discriminate between high drip and low drip phenotypes using a subset of 25 proteins with an accuracy of 63%. Models discriminating between divergent phenotypes with accuracy of 72% and 73% were also derived comparing respectively, high drip plus intermediate phenotype (considered as one phenotype) versus low drip and comparing low drip plus intermediate phenotype (considered as one phenotype) versus high drip. In all comparisons, the general classes of discriminatory proteins identified include metabolic enzymes, stress response, transport and structural proteins. In this research we have enhanced our understanding of the protein related processes underpinning this phenotype and provided strong data to work toward development of protein biomarkers for water holding capacity.

## Introduction

Increased *post mortem* degradation of muscle proteins has been associated with development of, and improvement in, key pork quality traits such as tenderness and water holding capacity (WHC) and also underpins the phenomenon of meat ageing. Numerous proteomic studies have shown that very many of the observed *post mortem* proteome changes relate to these protein degradation processes [1–4]. Other studies also highlight the roles of protein oxidation and programmed cell death in meat quality development [5–7].

WHC is a major quality attribute of fresh meat. The ability of pork to retain its water impacts the value, nutritional profile and consumer acceptance of pork meat [8,9]. WHC can be measured as drip loss and is influenced by many factors such as genotype, stress load before slaughter and pre-, peri- and post-slaughter interventions [10]. Factors that lead to variation in WHC in meat are known to influence the muscle/meat proteome [11–13]. For example, van de Wiel et al. [12] identified desmin as a potential marker protein for drip loss. Higher abundance of this protein was observed in samples with low drip loss. The rate of desmin degradation was correlated to the duration and degree of myofibril shrinkage and thereby to the phenomenon of drip loss.

The proteomes of pork loin samples with contrasting drip loss levels have been shown in previous studies [14,15] to differ significantly at the early *post mortem* timepoint of 1 day *post mortem*. It is not clear whether these physiologically divergent meat samples respond differently to the process of meat ageing and thus differ closer to the point of consumption, ideally 7 days *post mortem*. Indeed, in pale soft exudative (PSE) meat, inferior tenderness is often observed after ageing [16] and this is likely due to the interaction of physiology and environment to create physiological conditions that inhibit proteolysis of specific muscle proteins [17,18]. Therefore, a proteomic approach could offer insights on meat ageing in relation to WHC.

Proteomics has great potential to guide the discovery of biomarkers that can be used to reduce meat quality variability and facilitate management decisions. Biomarkers are biological indicators of some biological state or condition, often molecules such as proteins or metabolites aiming to predict the environmental effect on phenotype [13]. The identification of a subset of proteins (protein signatures) that have different abundance patterns across divergent phenotypes for WHC would be of benefit for meat processors, facilitating logistical decisions. In this study, two dimensional Difference Gel Electrophoresis (2-D DIGE) was used to investigate the changes in metabolic proteins in porcine centrifugal exudate that occur over the *post mortem* ageing period. L1-regularized logistic regression was mainly used to determine whether a subset of spots/proteins could be derived which would permit samples with different levels of drip loss to be discriminated and secondly improve our understanding of putative biomarkers of pork water holding capacity.

## Materials and Methods

### Animal sampling and meat quality measurements

The samples used in the current study were selected from a panel of 31 halothane free Large White x Landrace/Large White gilts with a similar genetic background aged six months. Animals were electrically stunned and slaughtered under controlled conditions at a live-weight of approximately 100 kg in an EU licensed pilot-scale abattoir at Teagasc, Food Research Centre Ashtown, Dublin. Several technological quality measurements were undertaken on the *longissimus thoracis et lumborum* (LTL) post slaughter, namely: pH, temperature, colour and drip loss. Loin pH and temperature were recorded from 45 min (pH _45_) up 24 h *post mortem* using a portable pH meter (Orion Research Inc., Boston, MA 02129, USA) and a pH electrode (pH/mV Sensors Ltd., Murrisk-Westport, Co. Mayo, Ireland), which was adjusted for muscle temperature before being inserted into muscles. Insertion point on the LTL was between the 10th and 11th rib. This enabled monitoring of the rate and extent of pH decline through to the ultimate pH (pH _u_) at 24 h *post mortem*. Colour was measured from a transverse section of the LTL muscle after 3 h blooming at 1, 3 and 7 days post slaughter using MiniScan XE Plus (Hunter Associates Laboratory, Inc., Reston, USA), with a D65 illuminant, 10° standard observer angle and 32 mm aperture size. Drip loss was determined according to the bag method of Honikel [19]. Briefly, samples (2.5 cm, 80 g) were removed from the LTL of each animal, suspended at 4°C for 48 h and reweighed. Drip loss was then expressed as a percentage of the original weight of the steak. Further details of the methodology for the technological quality measurements are provided in Di Luca *et al*., [14].

Based on criteria described in Di Luca *et al*., [14], samples were categorized in three phenotype classes based mainly on drip loss and pH values, termed high drip (HDrip), low drip (LDrip) and intermediate phenotype (IP). A full description of the parameters that allowed the identification of animals as PSE and dark, firm and dry (DFD) has been provided in Di Luca *et al*., [14]. Briefly, animals not displaying signs of PSE or DFD meat, but showing respectively high drip loss (drip loss ˃ 5%, pH _45_ ˃ 6.17) (HDrip) and low drip loss (drip loss ˂ 2.9%, pH _u_ ˂ 5.56) (LDrip) were selected to specifically examine divergence in drip loss. Samples with drip loss between 3.5 and 4.4%, pH _45_ ˃ 6.2 and pH _u_ ˂ 5.8 were selected as being representative of intermediate phenotype (IP). For each of the three quality phenotype of interest (HDrip, LDrip and IP) four animals were selected resulting in a total of 12 animals selected, from the initial 31, for this study.

### Exudate collection

From the 12 animals selected, four muscles displaying HDrip (drip loss > 5%, pH _45_ > 6.17, no PSE), four showing LDrip (drip loss < 2.9%, pH _u_ < 5.56, no DFD) and four IP (3.5 < drip loss < 4.4%, pH _45_ > 6.16 and pH _u_ < 5.80) at days 1, 3 and 7 *post mortem* (total of 36 exudate samples), centrifugal drip samples were collected. Exudate was collected from the muscle at days 1, 3 & 7 *post mortem* following a modified protocol of Bouton, Harris, and Shorthose [20], as reported in [14]. Briefly, three 8 g cores (12 mm diameter×2.5 cm) taken from slices of LTL muscle from each sample were centrifuged in polyalcomer centrifuge tubes (25×89 m; Beckman) for 60 min (Beckman Optima™ XL - 100K Ultracentrifuge, USA). After centrifugation, the exudate was snap frozen in liquid nitrogen and stored at −80°C until required. The protein concentration of all samples was determined in triplicate according to a modified Bradford assay protocol using a BSA standard [21].

### 2-D DIGE

Samples were compared in one experiment using 2-D DIGE (Ettan DIGE, Ge Healthcare, UK) as described in Di Luca *et al*., [15]. Samples used were muscle exudate collected from each of the three quality classes, HDrip, LDrip and IP (with four animals per class), at days 1, 3 and 7 *post mortem*; resulting in a total of 36 exudate samples. Using the minimal labelling technique [22], samples and internal standard were respectively labelled with Cy5 and Cy3 dye fluors (GE Healthcare, UK), according to the manufacturer’s instructions. Passive in-gel rehydration using immobilised DryStrips pH 4–7, 24 cm (GE Healthcare, UK) gradients [15], in which were loaded 50 μg of labelled sample proteins plus 50 μg of labelled pool proteins, was carried out overnight in the dark. The isoelectric focusing was performed using Ettan IPG Phor3 (GE Healthcare, UK) under the following conditions: 3500 V at 75000VHrs; gradient 8000 V for 10 min; 8000 V for 1Hour and holding step at 100 V. After isoelectric focusing IPG strips were reduced and then alkylated [15]. The proteins were further separated in the second dimension using a 12% SDS-PAGE gel at 15°C overnight in the dark by means of a PROTEAN Plus Dodeca Cell (Bio-Rad, Hercules, CA).

### Image analysis

The DIGE gels were scanned at 100 μm resolution using a Typhoon scanner 9200 (GE Healthcare, UK) at two different wavelengths (CyDye3, green laser 532 nm and CyDye5, red laser 633 nm). Two images per gel were obtained (72 in total). The scanned images were analyzed using Progenesis SameSpots (Nonlinear Dynamics, Durham, NC). Spots were both automatically and manually detected to avoid undetected or incorrectly detected spots. The protein spots detected in each image were automatically linked between the two images per gel. The most representative gel was selected as reference and then all the gels were matched to it. Following the spot detection and matching, spot volume were normalised and statistically analysed.

### Protein spot identification

Spots that were significantly different were matched with the ones successfully characterised by MALDI TOF/TOF or LTQ ORBITRAP XL in a previous paper [15].

Briefly, to allow an easier matching between DIGE gels and preparative gels (see below) and to allow sufficient peptides extraction for mass spectrometric analysis, preparative gels (from different phenotypes) were run loading four different amounts of protein (200 μg, 400 μg, 500 μg, 600 μg). The same separation conditions previously described for 2D DIGE were used. These gels were stained with a PlusOne silver stain kit (GEHealthcare, UK), compatible with downstream mass spectrometric analysis. The spots of interest identified by the DIGE study were matched to the silver stained gels, manually excised and in-gel digested with trypsin (Sequencing Grade Modified, Promega, Madison, NJ, USA).

MALDI-TOF mass spectrometric analysis was carried out with a 4800 plus MALDITOF/TOF Analyzer (Applied Biosystems, Foster City, CA, USA), as previously described [15]. The data obtained were screened against a porcine database (UniSprot-porcine; 06/11/09) and all entries database (Sprot: 14/12/09).

The spots for which an unambiguous identification could not be obtained by MALDI MS were re-analysed by nano-ESI LC-MS/MS.

A Thermo Scientific LTQ ORBITRAP XL mass spectrometer was connected to an Eksigent NANO LC.1DPLUS chromatography system incorporating an auto-sampler, as described [15]. TurboSEQUEST (Bioworks Browser 3.3.1 SP1; Thermo Scientific, UK) was used to search the porcine subset of the Uniprot Swissprot/Trembl fasta database (December 2009) and the Unprot/Swissprot database (March 2009) for fully and partially tryptic peptides.

Spots that were analysed by nano-ESI LC-MS/MS were processed using PEAKS Studio 6. Additional analysis using PEAKS Studio 6 identified a total of 22 new spots (S1 Fig) and associated proteins/peptides (S1 Table) available in the UCD-2DPAGE database (http://proteomics-portal.ucd.ie) under the filename ‘Porcine Database’.

### Differential abundance of protein spots

Following spot detection and matching across the 2-D DIGE gels, statistical analysis of the log standardized abundance changes between groups was performed using the software incorporated in Progenesis SameSpots. Principal Component Analysis (PCA) was applied as an exploratory data analysis tool to visualise differences between samples [three phenotypes (12 animals) studied over three timepoints, for a total of 36 samples] [23].

Moreover, to examine the clustering patterns in the proteome data for the three phenotypes over three timepoints, a hierarchical clustergram was obtained from all 376 normalised spots, which were log2 transformed and zero centred (the mean of each samples was subtracted from the datapoints for that sample). The hierarchical clustering used non-centred Pearson correlations as a pairwise similarity measure (puma.princeton.edu) [23].

### Identification of proteomic markers of drip loss

L1-regularized logistic regression [24] was carried out using l1-logreg software to determine if a subset of proteins could be identified whose abundance discriminated between drip loss sample groupings. This type of algorithm is able to extract relevant information from large datasets and point to a smaller number of highly significant biomarker proteins. This is a powerful discriminatory method that provides the explicit probabilities of classification apart from the class label information. In this method samples [three phenotypes (12 animals) studied over three timepoints, for a total of 36 samples] were separated into 2 groups in three comparisons [HDrip versus LDrip; LDrip plus IP (considered as one phenotype) versus HDrip; and HDrip plus IP (considered as one phenotype) versus LDrip]. Models were validated using ‘leave one out’ cross-validation resulting in explicit probabilities of classification and producing subsets of proteins discriminating conditions. The mean accuracy from cross validation was expressed as %.

### ANOVA

For comparison with findings of the l1-regularized regression, an ANOVA was carried out. The normalised volume of a spot was compared across groups using one way ANOVA (analysis of variance) p-value ≤ 0.05. The difference between phenotypes was expressed as fold change and calculated from the mean normalised volumes between the highest of the changes between the three phenotypes. The biological function of the proteins identified was obtained using PANTHER ontology analysis [25].

## Results and Discussion

Differences in WHC between pork carcasses has long been an economic problem for the meat industry [9,12,26,27]. With a view to addressing this issue, we undertook a proteomic approach. The data obtained in the current study parallel and are complementary to the findings of two recent papers which, along with this work, analysed a linked set of animals/tissues. Previous work presented the first 2DE-based protein map of porcine muscle exudate (centrifugal drip) and proteins/peptides associated with variability in exudate loss from pork meat at one day *post mortem* were identified [15]. Subsequently, the pathways and processes underlying the *post mortem* ageing period in intermediate (‘good quality’, IP) pork samples were identified, which showed the importance of *post mortem* modification and degradation of proteins in the development of meat quality [2]. Here, the *post mortem* protein abundance profiles (day 1, 3 and 7 *post mortem*) of samples with divergent levels of drip loss (HDrip, LDrip and IP) were analysed to determine firstly, the proteins that discriminate samples in relation to WHC using the relatively novel approach of l1-regularized logistic regression, secondly to investigate how drip loss biomarkers across WHC phenotypes evolve at days 3 and 7 *post mortem* and lastly, to determine if the ageing patterns differed among extremes of meat quality. Of particular note is that the approach taken across these three studies excluded the more well described DFD and PSE phenotypes from the sample set, hence directing the focus on the WHC trait uninfluenced by these conditions.

### Effect of ageing and phenotype on meat quality

Our interest was in understanding the unexplained cellular variations at the protein level, which contribute to variation in drip loss and for this reason we selected high and low drip loss samples but excluded specific pathological conditions with known causes i.e. pale soft and exudative (PSE-like) and dark, firm and dry (DFD-like) samples from the initial panel of divergent samples. We selected samples with no significant difference in pH _45_, pH _3_ and pH _u_ between the three (HDrip, LDrip, IP) quality classes we studied. This means they did not show any signs of PSE or DFD despite their high and low drip losses. Repeated measures ANOVA (p ≤ 0.05) revealed no significant difference in any other meat quality trait across the three phenotypes (Table 1) apart from the trait of interest, drip loss. Differences were however, observed across timepoints (p < 0.001). While the effect of ageing on important traits such as tenderness and colour was not affected by drip loss phenotype, we observed an interaction between ageing time and yellowness score (b*) (between days 1 and 7 and between days 3 and 7 *post mortem*) in all three phenotypes. This finding is in agreement with other studies [28,29]. Storage of pork influences colour and the ability of pork to bloom [28,30–32], which may relate to protein degradation processes *post mortem*.

**Table 1:** Mean ± standard error of meat quality traits relevant to WHC in the Large White x Landrace/Large White population across seven days *post mortem*.

Trait	HDrip samples (n = 4)	IP samples(n = 4)	LDrip samples(n = 4)
Drip loss (%)	6.1 ± 1.4	3.9 ± 0.4	2.5 ± 0.3
pH 45 min	6.3 ± 0.1	6.4 ± 0.2	6.5 ± 0.2
pH 3 h	5.9 ± 0.3	5.9 ± 0.2	5.9 ± 0.4
pH 24 h	5.6 ± 0.1	5.5 ± 0.1	5.5 ± 0.1
CIE L* (Day 1)	54.8 ± 3.2	55.4 ± 2	53.8 ± 4
CIE L* (Day 3)	54 ± 2.3	54.8 ± 2.6	54.6 ± 3.7
CIE L* (Day 7)	56.3 ± 3.9	54.4 ± 3.9	55.3 ± 3.5
CIE a* (Day 1)	8 ± 2.5	7.3 ± 0.7	7.7 ± 1.7
CIE a* (Day 3)	9.3 ± 3.9	9.6 ± 3.7	10.6 ± 4.8
CIE a* (Day 7)	8.8 ± 0.7	9.3 ± 2.1	8.9 ± 1.9
CIE b* (Day 1)	15.5 ± 0.6^*a*^	15.4 ± 0.5^*a*^	15.3 ± 0.7^*a*^
CIE b* (Day 3)	14.8 ± 0.8^*a*^	15.5 ± 0.5^*a*^	14.8 ± 1.3^*b*^
CIE b* (Day 7)	16 ± 1.1^*b*^	16.7 ± 1.1^*b*^	16.4 ± 1.4^*c*^
WBSF Day 1 (N)	46.1 ± 7.6^*a*^	45.7 ± 3.2^*a*^	40.2 ± 4.6^*a*^
WBSF Day 3 (N)	52.8 ± 10.7^*b*^	40.3 ± 5.2^*b*^	43.5 ± 7.2^*a*^
WBSF Day 7 (N)	37.9 ± 6.4^*c*^	32 ± 3.5^*c*^	31.8 ± 1.2^*b*^

HDrip, high drip loss; LDrip, low drip loss; IP, intermediate phenotype; L*, lightness; a*, redness; b*, yellowness; WBSF, Warner Bratzler shear force; N, Newtons. Within columns, for WBSF and CIE b* data, different italicised superscripts indicate significantly different means at the 5% level. Some values in this table were also presented in Table 1 of Di Luca *et al*., [14].

### Identification of protein spots using 2-D DIGE

**Fig 1:**
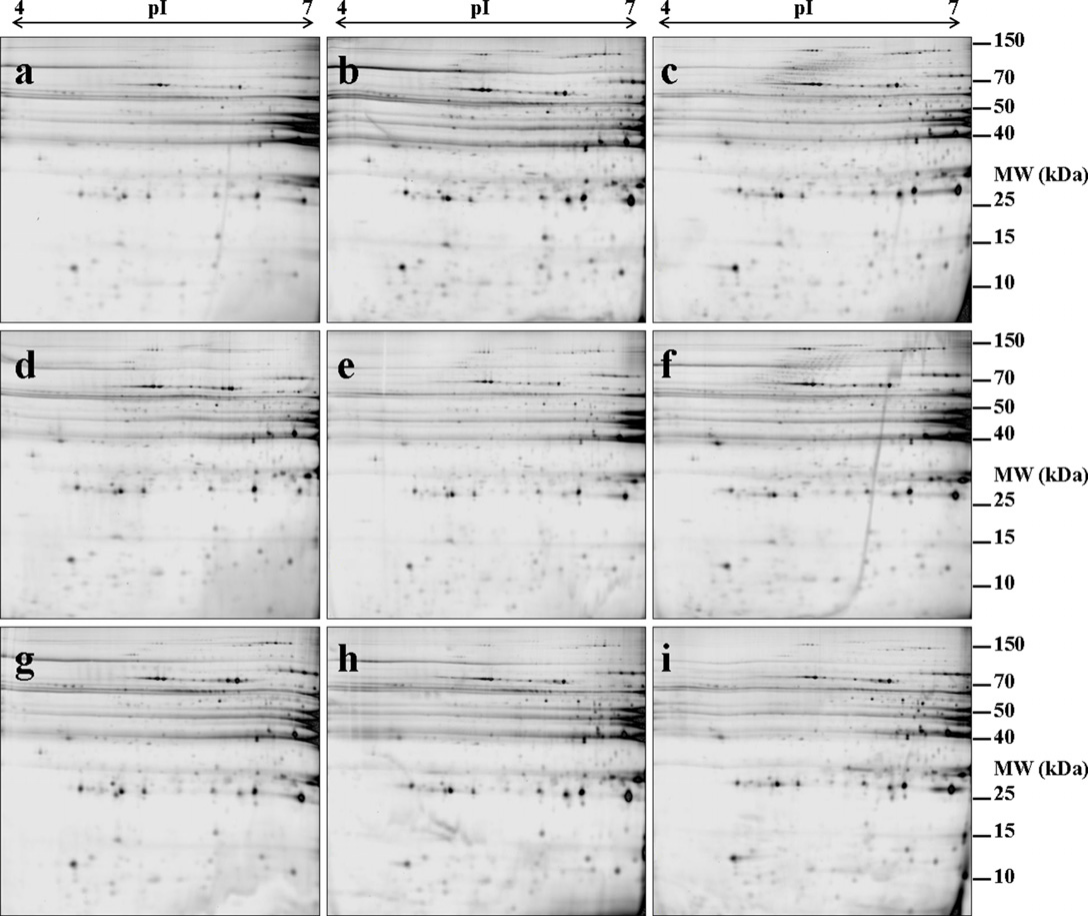
2-D DIGE gel images. Nine representative 2-D DIGE gel images that have been labelled with CyDye 5.

As previously reported [15], image analysis using Progenesis SameSpots identified 376 distinct protein spots in the centrifugal drip proteome of the samples under study. In our two previous studies, centrifugal drip proteome changes across three different phenotypes (HDrip, LDrip and IP) at day 1 *post mortem* were investigated using 12 samples i.e. n = 4 for each phenotype [15] and proteomic changes over three days *post mortem* in the IP phenotype was investigated using the 4 samples in this phenotype [2]. In the current study, samples from day 3 and 7 *post mortem* of the two divergent phenotypes (HDrip and LDrip) were also included, giving a total of three phenotypes divergent in WHC (HDrip, LDrip and IP) at three timepoints i.e. day 1, day 3 and day 7 *post mortem* (36 samples). Fig 1A to 1I shows representative gel images that have been labelled with CyDye 5 for the three phenotypes divergent in WHC (HDrip, LDrip and IP) at day 1, day 3 and day 7 *post mortem*.

Centrifugal drip proteins were separated by 2-D DIGE using immobilised pH 4–7 gradients (24 cm, linear) in the first dimension and 12% SDS-PAGE in the second dimension. Fig 1A, 1B and 1C show, gel images of low drip (LDrip) phenotype for WHC at days 1, 3 and 7, respectively. Fig 1D, 1E and 1F shows gel images of an intermediate phenotype (IP) for WHC respectively at days 1, 3 and 7. Fig 1G, 1H and 1I shows gel images of high drip (HDrip) phenotype for WHC respectively at days 1, 3 and 7.

### Protein identification

Eighty-nine spots were previously [15] characterised by MALDI TOF/TOF or LTQ ORBITRAP XL (Table 2). Thirty-six of these spots were found to be associated with meat ageing and/or drip loss (see below). These match 60 relevant proteins/peptides, with some of these proteins/peptides being identified in more than one spot and some spots containing more than one protein/peptide. As there were many spots that could not be identified following mass spectrometric data interpretation, we used PEAKS Studio 6 to increase the number of spots/proteins identified. This software has the capacity to integrate traditional database matching with *de novo* sequencing. Forty-two further spots were identified using this approach, matching 111 relevant proteins/peptides, with some of these proteins/peptides being identified in more than one spot and some spots containing more than one protein/peptide. These proteins are listed in S1 Table.

**Table 2:** Protein/fragment spots in porcine centrifugal drip that discriminate between HDrip and LDrip animals.

Spot^a^	Protein identified	Biological process ^b^	Spot ratios	
			HDrip	LDrip
**13**	Phosphoglucomutase-1	carbohydrate metabolic process	1.324	1.025
**13**	Stress induced phosphoprotein 1	immune system process; protein metabolic process; response to stress	1.324	1.025
**54**	Serum albumin	transport	0.943	1.281
**54**	Transferrin	macrophage activation; induction of apoptosis; cell surface receptor linked signal transduction; intracellular signaling cascade; cell-cell signalling; signal transduction	0.943	1.281
**56**	Heat shock protein 70	immune system process; protein metabolic process; response to stress	1.051	1.271
**65**	Filaggrin-2	protein metabolic process; cellular component morphogenesis; ectoderm development	1.125	0.979
**65**	Phosphatidylinositol 4,5-bisphosphate 3-kinase catalytic subunit gamma isoform	protein targeting; endocytosis; negative regulation of apoptosis; transmembrane receptor protein tyrosine kinase signaling pathway; phosphate metabolic processphospholipid metabolic process	1.125	0.979
**86**	Alpha-2-HS-glycoprotein (Fragment)	immune system process; protein metabolic process; mesoderm development; skeletal system development	0.504	0.373
**116**	Triosephosphate isomerase (Fragment)	fatty acid biosynthesis; gluconeogenesis; glycolysis	1.208	1.011
**116**	Phosphoglucomutase 1 (Fragment)	carbohydrate metabolic process	1.208	1.011
**218**	Serum albumin	transport	1.041	1.435
**218**	hemopexin	vitamin transport	1.041	1.435
**218**	myosin-1	muscle contraction; sensory perception; intracellular protein transport; mitosis; cytokinesis; cell motion	1.041	1.435
**218**	myosin-2	muscle contraction; sensory perception; intracellular protein transport; mitosis; cytokinesis; cell motion	1.041	1.435
**218**	myosin-7	muscle contraction; sensory perception; intracellular protein transport; mitosis; cytokinesis; cell motion	1.041	1.435
**239**	Hemopexin	vitamin transport	0.933	1.305
**239**	Phosphatidylinositol 3-kinase catalytic subunit type 3	protein targeting; endocytosis; negative regulation of apoptosis; transmembrane receptor protein tyrosine kinase signaling pathway; phosphate metabolic process	0.933	1.305
**277**	Serum albumin	transport	1.081	0.94
**277**	Beta-lactoglobulin	transport	1.081	0.94
**277**	hemopexin	vitamin transport	1.081	0.94
**277**	transthyretin	transport	1.081	0.94
**332**	Triosephosphate isomerase	fatty acid biosynthesis; gluconeogenesis; glycolysis	0.702	0.817
**375**	Vesicle-associated membrane protein-associated protein B	intracellular protein transport; vesicle-mediated transport; cell motion	0.914	1.164
**375**	Heat shock 70 kDa protein 1A	immune system process; protein metabolic process; response to stress	0.914	1.164
**375**	Heat shock 70 kDa protein 1-like	immune system process; protein metabolic process; response to stress	0.914	1.164
**375**	Heat shock 70 kDa protein 6	immune system process; protein metabolic process; response to stress	0.914	1.164
**648**	Phosphoglycerate kinase 1	carbohydrate metabolic process	0.948	1.16
**648**	Phosphoglycerate kinase 2	carbohydrate metabolic process	0.948	1.16
**772**	Proteasome subunit beta type-7	proteolysis	0.987	1.225
**1061**	Vimentin	ectoderm development; cellular component morphogenesis	0.946	1.232
**1061**	Filamin-A	protein localization at cell surface	0.946	1.232
**1061**	Actin, cytoplasmic 1	intracellular protein transport; exocytosis; endocytosis; mitosis; cytokinesis; cellular component morphogenesis	0.946	1.232
**1061**	Actin, alpha skeletal muscle	intracellular protein transport; exocytosis; endocytosis; mitosis; cytokinesis; cellular component morphogenesis	0.946	1.232
**1219**	Triosephosphate isomerase	fatty acid biosynthesis; gluconeogenesis; glycolysis	1.178	1.318
**1219**	Serum albumin	transport	1.178	1.318
**1219**	Creatine kinase M-type	muscle contraction; metabolic process	1.178	1.318
**1270**	Proteasome subunit beta type-6	protein metabolic process	1.403	1.288
**1270**	Translationally-controlled tumor protein	carbohydrate metabolic process	1.403	1.288
**1290**	Heat shock 70 kDa protein 1A	immune system process; protein metabolic process; response to stress	0.924	1.125
**1290**	Heat shock 70 kDa protein 6	immune system process; protein metabolic process; response to stress	0.924	1.125
**1290**	Serum albumin	transport	0.924	1.125
**1290**	Myc box-dependent-interacting protein 1	neurotransmitter secretion; intracellular protein transport; endocytosis; synaptic transmission; cell-cell signaling	0.924	1.125
**1290**	Heat shock 70 kDa protein 1B	immune system process; protein metabolic process; response to stress	0.924	1.125
**1290**	Heat shock 70 kDa protein 1-like	immune system process; protein metabolic process; response to stress	0.924	1.125
**1290**	Glucose-6-phosphate isomerase	gluconeogenesis; glycolysis	0.924	1.125

^a^Spot numbers refer to Fig 3 in our previous study [15].

^b^Biological process of the proteins obtained using PANTHER analysis [25].

### Principal component analysis (PCA) and hierarchical clustering

**Fig 2:**
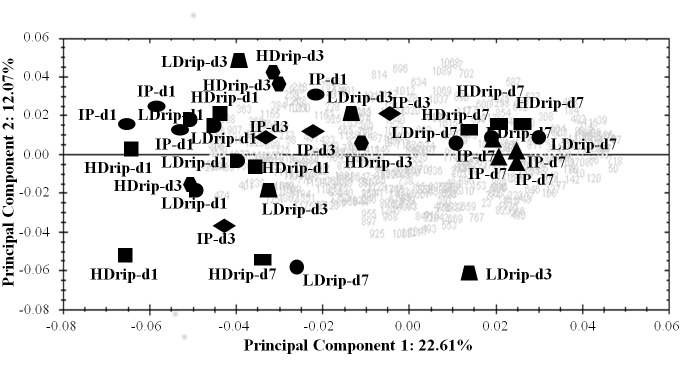
Principal Component Analysis (PCA). PCA biplot carried out using 376 spots from animals with divergent phenotypes for WHC (HDrip, high drip loss; IP, intermediate phenotype; LDrip, low drip loss; HDrip day 1; IP day 1; LDrip day 1; HDrip day 3; IP day 3; LDrip day 3; HDrip day 7; IP day 7; LDrip day 7) across the three days *post mortem*. Protein spots are represented by grey numbers. Distinct clustering of the samples by days *post mortem* is evident from the abundance of these proteins.

All identified spot variables were used to derive principal components for the different phenotypes (HDrip, LDrip and IP), over three days *post mortem*. The first principal component accounted for 22.61% of the variance and the second for 12.07% and the graph of these two PCs may be seen in Fig 2. When just the IP phenotype was examined in a previous paper [2], the first principal component showed clear structuring across day of sampling *post mortem* [2]. Here, even when contrasting phenotypes were included together, the first principal component still primarily structured centrifugal drip samples based on *post mortem* day of sampling and, in all likelihood, the ageing process. Samples of all three phenotypes (HDrip, LDrip and IP) from day 1 and day 7 represent the extremes, with day 3 samples falling in between. Clear separation based on drip loss phenotype using all 376 variables (all spots detected) was not evident from either of the first two principal components. However, within the day 7 cluster, HDrip samples were somewhat contrasted to IP samples by the second principal component (Fig 2), suggesting that by the end of the ageing period, at the time of likely consumption of the product, the proteomes do diverge somewhat. It can also be seen in Fig 2 that a high number of protein spots (grey) co-localise with samples representing day 7 *post mortem* of all three phenotypes, indicating they are most abundant at this timepoint, and thus accumulate over the ageing process.

**Fig 3:**
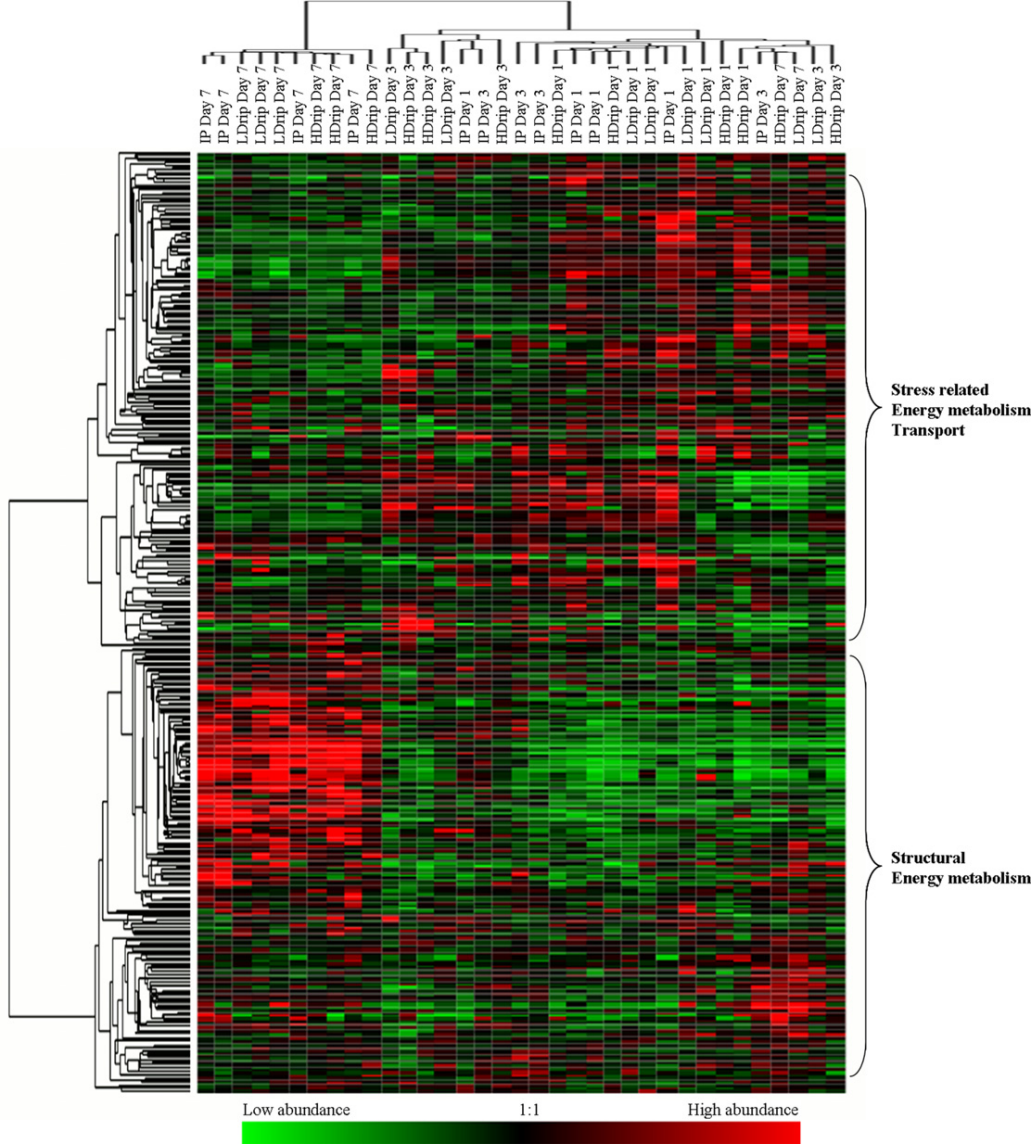
Hierarchical clustergram. It can be seen from Fig 3 that there is a clear separation of samples based on the day of measurement. The major split in the protein profiles is between proteins that are more abundant at day 1 and 3 and those that are more abundant at day 7. The next greatest split separates those proteins which are more abundant at day 1 from those more abundant at day 3. According to the spots that have been identified by mass spectrometry [S1 Table and Table 2 in Di Luca *et al*., [15]], those more abundant at day 1 and 3 were mainly stress related, energy metabolism and transport proteins, whereas those more abundant at day 7 were mainly structural and energy metabolism proteins. The clustergram does not show clear separation of the three phenotypes (HDrip, LDrip and IP) within individual timepoints.

A hierarchical clustergram of spots/proteins and samples may be seen in Fig 3, constructed using all observed spot variables (n = 376) for the three different phenotypes (HDrip, LDrip and IP) over three days *post mortem* studied. Higher abundance is indicated by red colour, with lower abundance indicated by green colour. Proteins with similar abundance profiles across the dataset cluster together, as do samples with similar proteomic profiles.

Both PCA and the hierarchical clustergram suggest that the major patterns in protein abundance data closely reflect the *post mortem* ageing process. *Post mortem* protein degradation contributes to the development of meat quality traits [4,33,34]. The rate and extent of protein degradation differs from protein to protein [35–37]. These differences have been associated with quality traits such as WHC and tenderness. In this study, it is difficult to observe a clear separation between the three different drip loss phenotypes based on the PCA and clustergram analyses. No real structuring according to phenotype is observed at early timepoints, but this change by day 7 *post mortem*, when samples are better clustered by their drip loss level. This suggests that the physiological differences in drip loss may manifest themselves in the proteome at later timepoints *post mortem*. The bi-plots show that the abundance of many spots in the study is positively correlated with day 7 samples. Similar data were observed in the clustergram, where a higher number of spots/proteins were observed to be more abundant (in red) at day 7.

Matching the spots identified by mass spectrometry with the spots/proteins in the clustergram, the main class of proteins identified in that region of the clustergram are structural proteins (e.g. titin, vinculin, myosin). These likely are protein fragments as a result of proteolytic activity [1]. The main classes of proteins identified to be more abundant in the samples from day 1 *post mortem*, are stress related, energy metabolism and transport proteins such as protein DJ-1, peroxiredoxin 2, phosphoglucomutase 1 (PGM1), protein CutA, etc. This group of proteins declines in abundance over the *post mortem* period. Several glycolytic enzymes (e.g. enolase and triosephosphate isomerase) in particular show high abundance at day 1 followed by a decline over the *post mortem* ageing period. The might be due to the degradation of these proteins, as observed elsewhere [1,38]. Heat shock proteins (HSPs) such as HSP70, αβ-crystallin have a protective role and may slow down the process of cellular death [39,40]. Similar classes of proteins were observed in the small cluster of spots/proteins more abundant at day 3, which may indicate that some of these proteins may still exert their protective functions after several days *post mortem*.

### Identifications of proteins that discriminate samples in relation to WHC

Most of the water in *post mortem* muscle is entrapped in the myofibrillar structures of the cell. Alterations of these structures e.g. due to early *post mortem* events such as the rate and extent of pH decline, proteolysis and protein oxidation will influence the ability of meat to retain water [9,41]. The identification of a subset of proteins that have a different abundance pattern across divergent phenotypes for WHC would both enhance our scientific understanding of the phenotype and be of benefit for meat processors. In proteomics, a classical approach to discriminate groups of samples from the large amount of data generated (spots/proteins abundance) is univariate data analysis (e.g. one way ANOVA, Student t-test). However, univariate data analysis is a limited approach to extract meaningful information from the complex data generated from proteomic approaches. Multivariate data analysis approaches, such as partial least squares regression, have been developed to interpret the relationship between the different proteins [42]. Unlike univariate data analysis, they are particularly useful at examining datasets with multi co-linearity (e.g. spots which act in concert with one another) and detect underlying trends [43].

The large amount of data obtained (376 spots from each samples across timepoints) was analysed using l1-regularized logistic regression [24], a machine learning algorithm that is able to extract relevant information from large datasets and point to a smaller number of highly significant biomarker proteins. This is a powerful discriminatory method that provides the explicit probabilities of classification apart from the class label information. This type of algorithm is well-suited to applications where the number of variables is much larger than the number of samples (e.g. microarray) [44]. Similar statistical models have been mainly used in clinical studies for early detection and diagnosis. In those settings, datasets for logistic regression analysis are usually obtained with a high throughput proteomics platform reaching high sensitivity, over 90%, i.e. mass spectrometry; [45–47]. It is known that 2D PAGE is a low throughput approach, and usually the amount of data generated with 2D PAGE is not sufficient for this approach. Here, through the use of 2D DIGE technology, the amount of data generated was adequate for this statistical approach to be applied. However, in order to apply l1-regularized logistic regression to identify a subset of spots/proteins that would permit discrimination of samples with different levels of drip loss, we had to combine data from all days *post mortem* (HDrip at days 1, 3 and 7; IP at days 1, 3 and 7; LDrip at days 1, 3 and 7) for each phenotype. We were also interested to segregate HDrip from all other samples, and LDrip from all other samples. Hence, we further combined phenotypes (LDrip plus IP considered as one phenotype and HDrip plus IP considered as one phenotype) to facilitate this additional comparison. Indeed, ‘Leave one out’ cross-validation of the l1-regularized logistic regression models revealed a degree of variation in the accuracy of the discriminatory models. Using this method 25 protein spots were identified which are able to discriminate between HDrip and LDrip samples with 63% accuracy, moreover in two other comparisons 25 and 22 spots discriminated between LDrip plus IP (considered as one phenotype) versus HDrip and between HDrip plus IP (considered as one phenotype) versus LDrip with 73% and 72% of accuracy, respectively. It was notable that when two phenotypes were combined (increasing the number of variables and reducing the number of conditions), improved discrimination was achieved (see below), suggesting that this approach has the capacity to be even more powerful in the search for biomarkers of meat quality, when a higher throughput of data is applied.

The full list of spots identified in this analysis is shown in Tables 2–4, together with their biological process identified using PANTHER analysis [25]. Seventeen of the 25 spots used to discriminate HDrip from LDrip samples were characterised by mass spectrometry, and 45 proteins/peptides were identified (Table 2). Forty-one proteins/peptides were identified in 14 of the 25 spots/proteins that discriminated HDrip from LDrip plus IP (Table 3). Forty-eight proteins/peptides were identified in 15 of the 22 spots/proteins that discriminated LDrip versus HDrip plus IP (Table 4). Several spots overlapped between the different comparisons.

**Table 3:** Protein/fragment spots in porcine centrifugal drip that discriminate between HDrip versus the LDrip plus IP animals.

Spot^a^	Protein identified	Biological process^b^	Spot ratios	
			HDrip	LDrip plus IP
**54**	Serum albumin	transport	0.943	1.278
**54**	Transferrin	macrophage activation; induction of apoptosis; cell surface receptor linked signal transduction; intracellular signaling cascade; cell-cell signalling; signal transduction	0.943	1.278
**56**	Heat shock protein 70	immune system process; protein metabolic process; response to stress	1.051	1.28
**90**	Peroxiredoxin-2 (Fragment)	immune system process; oxygen and reactive oxygen species metabolic process	1.094	1.249
**91**	Heat shock 70 kDa protein 1-like	immune system process; protein metabolic process; response to stress	0.884	0.94
**91**	Heat shock 70 kDa protein 6	immune system process; protein metabolic process; response to stress	0.884	0.94
**91**	Heat shock 70 kDa protein 1A	immune system process; protein metabolic process; response to stress	0.884	0.94
**94**	Proteasome subunit alpha type-5-A	protein metabolic process	0.976	0.881
**95**	Vitamin D(3) 25-hydroxylase	iron ion binding	0.915	1.069
**375**	Vesicle-associated membrane protein-associated protein B	intracellular protein transport; vesicle-mediated transport; cell motion	0.914	1.153
**375**	Heat shock 70 kDa protein 1A	immune system process; protein metabolic process; response to stress	0.914	1.153
**375**	Heat shock 70 kDa protein 1-like	immune system process; protein metabolic process; response to stress	0.914	1.153
**375**	Heat shock 70 kDa protein 6	immune system process; protein metabolic process; response to stress	0.914	1.153
**566**	Heat shock 70 kDa protein 1A	immune system process; protein metabolic process; response to stress	0.925	1.207
**566**	Heat shock 70 kDa protein 1-like	immune system process; protein metabolic process; response to stress	0.925	1.207
**566**	Heat shock protein 68	immune system process; protein metabolic process; response to stress	0.925	1.207
**566**	Heat shock 70 kDa protein	immune system process; protein metabolic process; response to stress	0.925	1.207
**566**	Heat shock cognate 71	immune system process; protein metabolic process; response to stress	0.925	1.207
**566**	Luminal-binding protein 2	immune system process; protein metabolic process; response to stress	0.925	1.207
**566**	Heat shock 70 kDa protein 1B	immune system process; protein metabolic process; response to stress	0.925	1.207
**566**	Heat shock 70 kDa protein 6	immune system process; protein metabolic process; response to stress	0.925	1.207
**566**	Serum albumin	transport	0.925	1.207
**576**	Peroxiredoxin-2 (Fragment)	immune system process; oxygen and reactive oxygen species metabolic process	1.288	1.582
**772**	Proteasome subunit beta type-7	proteolysis	0.987	1.172
**1000**	Beta-tropomyosin	structural constituent of cytoskeleton; muscle contraction; cell motion; cellular component morphogenesis	1.312	1.207
**1000**	Tropomyosin alpha-1 chain	muscle contraction; cell motion; cellular component morphogenesis	1.312	1.207
**1000**	Tropomyosin alpha-4 chain	muscle contraction; cell motion; cellular component morphogenesis	1.312	1.207
**1000**	Tropomyosin alpha-3 chain	muscle contraction; cell motion; cellular component morphogenesis	1.312	1.207
**1135**	Beta-enolase	glycolysis	0.949	1.168
**1192**	Heat shock 70 kDa protein 1A	immune system process; protein metabolic process; response to stress	1.054	1.328
**1192**	Serum albumin	transport	1.054	1.328
**1192**	Heat shock 70 kDa protein 6	immune system process; protein metabolic process; response to stress	1.054	1.328
**1192**	Heat shock 70 kDa protein 1B	immune system process; protein metabolic process; response to stress	1.054	1.328
**1192**	Heat shock 70 kDa protein 1-like	immune system process; protein metabolic process; response to stress	1.054	1.328
**1290**	Heat shock 70 kDa protein 1A	immune system process; protein metabolic process; response to stress	0.924	1.065
**1290**	Heat shock 70 kDa protein 6	immune system process; protein metabolic process; response to stress	0.924	1.065
**1290**	Serum albumin	transport	0.924	1.065
**1290**	Myc box-dependent-interacting protein 1	neurotransmitter secretion; intracellular protein transport; endocytosis; synaptic transmission; cell-cell signaling	0.924	1.065
**1290**	Heat shock 70 kDa protein 1B	immune system process; protein metabolic process; response to stress	0.924	1.065
**1290**	Heat shock 70 kDa protein 1-like	immune system process; protein metabolic process; response to stress	0.924	1.065
**1290**	Glucose-6-phosphate isomerase	gluconeogenesis; glycolysis	0.924	1.065

^a^Spot numbers refer to Fig 3 in our previous study [15].

^b^Biological process of the proteins obtained using PANTHER analysis [25].

**Table 4:** Protein/fragment spots in porcine centrifugal drip that discriminate between LDrip versus HDrip plus IP animals.

Spot^a^	Protein identified	Biological process^b^	Spot ratios	
			LDrip	HDrip plus IP
**92**	Heat shock 70 kDa protein 1A	immune system process; protein metabolic process; response to stress	1.252	1.046
**92**	Heat shock 70 kDa protein 6	immune system process; protein metabolic process; response to stress	1.252	1.046
**92**	78 kDa glucose-regulated protein homolog	response to stress	1.252	1.046
**92**	Luminal-binding protein	immune system process; protein metabolic process; response to stress	1.252	1.046
**92**	Heat shock 70 kDa protein 1B	immune system process; protein metabolic process; response to stress	1.252	1.046
**92**	Heat shock 70 kDa protein 1-like	immune system process; protein metabolic process; response to stress	1.252	1.046
**92**	Heat shock 70 kDa protein (Fragment)	immune system process; protein metabolic process; response to stress	1.252	1.046
**93**	Serum albumin	transport	1.436	0.926
**93**	Hemopexin	vitamin transport	1.436	0.926
**109**	Stress induced phosphoprotein 1	immune system process; protein metabolic process; response to stress	0.911	1.444
**170**	Serum albumin	transport	1.389	1.113
**170**	Glucose-6-phosphate isomerase	gluconeogenesis; glycolysis	1.389	1.113
**177**	Heat shock 70 kDa protein 1A	immune system process; protein metabolic process; response to stress	1.152	0.789
**177**	Vinculin	cell adhesion; cell motion; intracellular signaling cascade; cellular component morphogenesis	1.152	0.789
**177**	Heat shock 70 kDa protein 1B	immune system process; protein metabolic process; response to stress	1.152	0.789
**177**	Heat shock 70 kDa protein 6	immune system process; protein metabolic process; response to stress	1.152	0.789
**177**	Heat shock 70 kDa protein 1-like	immune system process; protein metabolic process; response to stress	1.152	0.789
**218**	Serum albumin	transport	1.435	0.975
**218**	Hemopexin	vitamin transport	1.435	0.975
**218**	Myosin-1	muscle contraction; sensory perception; intracellular protein transport; mitosis; cytokinesis; cell motion	1.435	0.975
**218**	Myosin-2	muscle contraction; sensory perception; intracellular protein transport; mitosis; cytokinesis; cell motion	1.435	0.975
**218**	Myosin-7	muscle contraction; sensory perception; intracellular protein transport; mitosis; cytokinesis; cell motion	1.435	0.975
**277**	Serum albumin	transport	0.94	1.074
**277**	Beta-lactoglobulin	transport	0.94	1.074
**277**	Hemopexin	vitamin transport	0.94	1.074
**277**	Transthyretin	transport	0.94	1.074
**326**	Phosphoglucomutase 1	carbohydrate metabolic process	1.577	1.084
**332**	Triosephosphate isomerase	fatty acid biosynthesis; gluconeogenesis; glycolysis	0.817	0.709
**503**	Beta-lactoglobulin	transport	1.153	0.999
**503**	Hemopexin	vitamin transport	1.153	0.999
**503**	Serum albumin	transport	1.153	0.999
**772**	Proteasome subunit beta type-7	proteolysis	1.225	1.055
**1061**	Vimentin	ectoderm development; cellular component morphogenesis	1.232	0.913
**1061**	Filamin-A	protein localization at cell surface	1.232	0.913
**1061**	Actin, cytoplasmic 1	intracellular protein transport; exocytosis; endocytosis; mitosis; cytokinesis; cellular component morphogenesis	1.232	0.913
**1061**	Actin, alpha skeletal muscle	intracellular protein transport; exocytosis; endocytosis; mitosis; cytokinesis; cellular component morphogenesis	1.232	0.913
**1219**	Triosephosphate isomerase	fatty acid biosynthesis; gluconeogenesis; glycolysis	1.318	1.205
**1219**	Serum albumin	transport	1.318	1.205
**1219**	Creatine kinase M-type	muscle contraction; metabolic process	1.318	1.205
**1270**	Proteasome subunit beta type-6	protein metabolic process	1.288	1.379
**1270**	Translationally-controlled tumor protein	carbohydrate metabolic process	1.288	1.379
**1290**	Heat shock 70 kDa protein 1A	immune system process; protein metabolic process; response to stress	1.125	0.967
**1290**	Heat shock 70 kDa protein 6	immune system process; protein metabolic process; response to stress	1.125	0.967
**1290**	Serum albumin	transport	1.125	0.967
**1290**	Myc box-dependent-interacting protein 1	neurotransmitter secretion; intracellular protein transport; endocytosis; synaptic transmission; cell-cell signaling	1.125	0.967
**1290**	Heat shock 70 kDa protein 1B	immune system process; protein metabolic process; response to stress	1.125	0.967
**1290**	Heat shock 70 kDa protein 1-like	immune system process; protein metabolic process; response to stress	1.125	0.967
**1290**	Glucose-6-phosphate isomerase	gluconeogenesis; glycolysis	1.125	0.967

^a^Spot numbers refer to Fig 3 in our previous study [15].

^b^Biological process of the proteins obtained using PANTHER analysis [25].

Many proteins that permit the discrimination of samples with different levels of drip loss, (e.g. stress induced phosphoprotein 1, triosephosphate isomerase, transferrin) were also highlighted as potential biomarkers for WHC in our previous paper [15].

In this study, the proteins identified in the three comparisons, are generally involved in stress response, energy metabolism and as structural components of the cell. As previously observed [2,15] and as expected with an exudate substrate, soluble proteins are the dominant classes. Stress related proteins are the most abundant proteins (mainly with a molecular weight of 70 kDa), especially in the two comparisons with the highest level of accuracy (a higher number of samples were included) where we segregated HDrip or LDrip from all other samples. Stress related proteins are known as heat shock proteins (HSP) and are divided into families according to their average molecular weight. The 70 kDa HSP family is composed of heat inducible proteins (HSP70), which are expressed under cellular stress conditions, and heat shock cognate proteins (HSC70), which are constitutively expressed without any stress stimulation [48]. These ATP-dependent proteins are involved in important cellular functions. HSC70 is most efficient when associated with heat shock factor cochaperones, protecting, preserving or recovering the proper functional conformation of proteins. Many proteomic studies in meat science have reported HSPs as potential biomarkers for several meat quality traits (WHC, tenderness, colour and flavour) [49–54]. Interestingly, all the HSPs identified in this study with the exception of spot 13 in the comparison between HDrip versus LDrip and spot 109 in the comparison between LDrip versus HDrip plus IP showed a higher abundance in the LDrip or LDrip plus IP compared to HDrip. Moreover, it is of interest that in both spots (13 and 109), stress-induced phosphoprotein 1 (STIP1) was identified and that following Western blot validation (two proteins were identified in spot 13) in Di Luca *et al*., [15], STIP1 was higher in abundance in the LDrip phenotype compared to both, HDrip and IP. It is known that HSPs are constitutively expressed but are also synthesised in response to growth, development and differentiation and in response to stresses [52,55]. A putative mechanism for the role of heat shock proteins in drip loss is that these proteins are protective against the cell-disrupting effects of stress and cell death, which lead to a loss of fluid from the cells and where they are in higher abundance, there is less drip lost. Moreover, these proteins move from the cytoplasm to the nucleus under stressed conditions [56,57]. The lower or higher abundance of these proteins in the centrifugal drip may be due to changes in solubility of the proteins. It could be hypothesized that due to a higher stress condition in the HDrip phenotype, HSPs could be localized in the nucleus (lower abundance in the centrifugal drip) whereas in the LDrip phenotype the presence of these proteins could be still high in the cytoplasm.

Several myofibrillar proteins like myosin, vinculin, actin were identified. Cytoskeletal proteins are components of the myofibrillar complex that provide support for the contractile and regulatory proteins and serve to stabilise the contractile apparatus. Myosin is the most abundant of the myofibrillar proteins [58]. It has been shown that the denaturation of myobrillar proteins, particularly myosin, is associated with the low WHC of PSE pork [59]. PSE meat compared with normal pork meat shows both reduced myofibrillar protein solubility and myosin ATP-ase activity, both are indicators of denaturation [60,61]. It is known that these proteins are targets of proteolytic enzymes in *post mortem* muscle [50,62], and a lower abundance of these proteins in our study in the HDrip phenotype could indicate an alteration of structural components in the myofibril which affects WHC [63]. On the other hand, some proteins like vinculin were identified in a region of the gel closer to the theoretical molecular weights of these proteins, which makes it more difficult to interpret the data. Fragments may start to accumulate with a molecular weight very close to the full protein as showed in Di Luca *et al*., [2]. Other minor proteins of the thin filaments such as tropomyosins (TPM) were identified (spot 1000). These proteins were identified in a spot close to the predicted location of the parent protein. In our previous study [15], TPM1 and TPM2 were identified in two other spots with a slightly different molecular weight and/or pI from that predicted, probably due to being electrophoretic variants or isoforms of these proteins. Two different patterns were observed for the two TPMs in Di Luca *et al*., [15], here a higher abundance of tropomyosins was observed in the HDrip phenotype compared to LDrip. Tropomyosins are substrates of μ-calpain under *post mortem* conditions [64] and have been shown to denature in low drip samples [65]. As the proteins identified in current study were identified in a spot close to the parent protein this may allude to a major degradation of the parent proteins and an accumulation of fragments of a lower molecular weight in the resultant gel.

Although myofibrillar protein denaturation contributes to the low WHC of PSE meat, it cannot completely explain the low WHC of meat that is not PSE but present high loss of water [59,66]. A role for sarcoplasmic proteins has been postulated to explain the low WHC in samples that are not PSE, as their denaturation is associated with low WHC in meat [59,66]. In the current study, several sarcoplasmic proteins like triosephosphate isomerase were identified. This protein was previously [15] highlighted as potentially predictive of drip loss. Moreover, precipitation of such protein has been related to PSE muscle and pork colour [67,68]. In this study, several spots showed the presence of triosephosphate isomerase (spots 116, 332 and 1219 in the comparison between HDrip versus LDrip; spots 332 and 1219 in the comparison between LDrip versus HDrip plus IP). The spots were identified in different pI and/or molecular weight, suggesting that they may represent multiple isoforms, fragments, crossed linked or PTM of the protein. Spots 332 and 1219 showed a higher abundance in the LDrip phenotype, whereas spot 116 in HDrip. In previous work [15], this protein was identified in a different spot (66) which was higher in abundance in the LDrip compared to HDrip phenotype. The difference in abundance pattern observed in spot 116 may be driven by the presence of another protein (phosphoglucomutase 1) in the same spot. Triosephosphate isomerase is a glycolytic enzyme and displayed a lower abundance in muscle with HDrip phenotype, possibly leading to a reduction in levels of ATP at early times *post mortem*. Once the sources of energy are exhausted, proteins will tend to denature, and thus are more liable to proteolysis [69], processes that may affect WHC.

It is of interest that as in [15], spot 54 (serum albumin and transferrin) is part of the signatures that are able to discriminate between HDrip versus LDrip and between HDrip versus LDrip plus IP. In both comparisons, the abundance of spot 54 is lower in the HDrip phenotype. A similar pattern was observed previously [15] where Western blot also showed that transferrin drove the change in the spots. Transferrins are glycoproteins which reversibly bind iron and help control free iron levels [70]; upregulated expression of this protein has been observed in hypoxic condition [71]. The higher abundance of transferrins in LDrip was highlighted as indicative of a differential muscle response to hypoxic situations which is linked to the manifestation of a quality trait such as drip loss [15].

Taken together, our finding highlights l1-regularized logistic regression as a powerful discriminatory method, moreover, a number of proteins were identified in both this and an earlier study which corroborates our findings.

### Comparison with analysis of variance

One way ANOVA (p ≤ 0.05) analyses were performed to reveal spots that were significantly different across the three phenotypes at days 3 and 7 *post mortem* [day 1 was covered in [15]]. The outcomes were then compared with multivariate clustering and logistic regression approaches.

Ten and 18 significant (p ≤ 0.05) spots whose abundance changed significantly across the three divergent WHC phenotypes at day 3 and 7 *post mortem* respectively were identified. Details of the significant spots at day 3 and 7 *post mortem* (Tukey Kramer post hoc analysis) are presented in Table 5A and 5B. Fig 3A of Di Luca *et al*., [15] and S1 Fig shows the location of the spots characterised by mass spectrometric analysis. From the ten spots/proteins that were observe to vary significantly (p ≤ 0.05) in abundance across phenotypes at day 3 *post mortem*, three spots/proteins were identified by mass spectrometry and three proteins/peptides were identified. On the other hand, 26 proteins/peptides were identified in eight of the 18 spots/proteins that were significantly changing at day 7 *post mortem*. S2 Table shows the biological process of the proteins identified [25]. Of the spots characterized by mass spectrometry, spot 109 in which STIP1 was identified was in common at day 3 and 7 *post mortem*. No spots identified at day 3 and 7 *post mortem* were matching with the spots identified in our previous study at day 1 *post mortem* [15]. In addition, six of the ten spots characterised at days 3 and 7 *post mortem* and four of the 14 spots characterised at day 1 *post mortem* [15] overlapped with those identified using the logistic regression approach.

**Table 5:** ANOVA p value, fold changes (calculated from the mean normalised volumes between the groups that shows the maximum of the changes) and average normalised spot volumes (Progenesis SameSpots) of the 10 (A) and 18 (B) spots significantly different (p ≤ 0.05) respectively at day 3 (A) and day 7 (B) *post mortem* between the three divergent phenotypes for WHC (HDrip, LDrip and IP).

**Spot**^a^	**ANOVA (p)**	**Fold change**	**(A) Mean normalised volume at day 3 *post mortem***		
			**HDrip Day 3**	**IP Day 3**	**LDrip Day 3**
**176**	0.045	1.9	0.522^a^	0.887^a^	0.996^a^
**109**	0.031	1.9	0.975^a^	1.818^b^	1.058^a^
710	0.014	1.8	0.983^a^^@^	0.543^b^^@^	0.916^a^^b^
362	0.048	1.7	0.991^a^^b^	1.243^a^	0.738^b^
265	0.003	1.6	1.019^a^^b^	1.241^a^	0.755^b^
**101**	0.025	1.5	1.07^a^	1.63^b^	1.12^a^
216	0.045	1.5	0.747^a^^b^	0.945^a^	0.641^b^
1077	0.015	1.4	1.025^a^	1.215^a^^b^	1.423^b^
1248	0.006	1.3	1.233^a^	1.292^a^	0.96^b^
785	0.02	1.3	0.976^a^	1.25^b^	1.249^b^
**Spot**^a^	**ANOVA (p)**	**Fold change**	**(B) Mean normalised volume at day 7 *post mortem***		
			**HDrip Day 7**	**IP Day 7**	**LDrip Day 7**
**177**	0.002	2.1	0.915^a^	0.699^a^	1.456^b^
893	0.014	2	0.87^a^	1.34^b^	0.683^a^
458	0.045	1.9	1.129^a^^b^	1.65^a^	0.887^b^
**326**	0.017	1.8	1.061^a^^b^	0.741^a^	1.338^b^
**109**	0.004	1.7	1.172^a^	1.36^a^	0.782^b^
**652**	0.04	1.7	0.807^a^^@^	1.287^b^^@^	0.763^a^
**116**	0.014	1.7	1.231^a^^b^	1.472^a^	0.877^b^
**935**	0.019	1.7	1.003^a^^b^	0.71^a^	1.173^b^
**1061**	0.048	1.6	1.12^a^^b^	1.014^a^^@^	1.64^b^^@^
143	0.04	1.5	0.762^a^^@^	1.173^b^^@^	1.02^a^^b^
1141	0.038	1.5	0.736^a^^@^	0.487^b^^@^	0.536^a^^b^
551	0.018	1.5	0.967^a^	1.37^b^^@^	1.403^b^
247	0.02	1.4	1.105^a^	0.766^b^	0.763^b^
**566**	0.007	1.4	1.15^a^	1.647^b^	1.328^a^^@^
398	0.035	1.3	0.819^a^^b^	0.772^a^	1.039^b^
1077	0.011	1.3	1.008^a^^@^	0.811^b^	1.074^a^
785	0.037	1.1	1.011^a^^b^	0.981^a^	1.127^b^
916	0.013	1.1	1.076^a^	1.132^a^^b^	1.21^b^

Within rows, different superscripts indicate significantly different means at the 5% level (following Tukey Kramer post hoc analysis).

^@^ indicates that the p-value was below 0.1.

^a^Spot numbers refer to Fig 3 in [15].

Spots number in bold are those identified by mass spectrometry.

Similarly to the multivariate approaches, the ANOVA revealed that the proteins associated with drip loss at day 3 and 7 *post mortem* can be generally categorised as stress response proteins, metabolic enzymes and structural proteins. Stress-related were the dominant class, suggesting that some of these proteins may still exert their protective functions after several days *post mortem*. Of the ten spots characterised by mass spectrometry in the two comparisons, several spots/proteins like STIP1, HSP70 were also highlighted as potential biomarkers for WHC using l1-regularized logistic regression and also in our previous study [15]. In addition, the majority of the proteins identified in the other four spots that were not overlapping, were also identified in other spots using l1-regularized logistic regression, highlighting again the strength of this approach. In contrast to the larger panel of marker proteins identified using logistic regression, only two spots characterised by mass spectrometry were significantly changing between HDrip and LDrip (spots 109 and 177 in the comparison at day 7 *post mortem*) by ANOVA. STIP1 was identified in spot 109, whereas in spot 177 were identified four stress related proteins and vinculin. Interestingly, the spot clustering in the PCA and in the hierarchical clustergram show a similar pattern across these phenotypes at day 7 *post mortem*. The comparison would suggest that the logistic regression approach may be more powerful than ANOVA, where data is sufficient to apply it.

### Utility of identified biomarkers

The research presented in this paper and the previous studies [2,15] provide substantial and novel data which greatly add to our body of knowledge of the water holding capacity phenotype in pork. The value of using a multivariate approach such as l1-regularized logistic regression has been clearly demonstrated. Our innovative approach of using 2D DIGE and l1-regularized logistic regression has enabled identification of protein signatures that are able to discriminate between divergent WHC phenotypes. Indeed, it has been shown that a combination of specific proteomic biomarkers shows higher accuracy than a single protein or nonspecific protein combinations [13]. While practical implementation of biomarkers for quality would ideally require just a single timepoint measurement, our data furthers the potential in this regard by demonstrating the key pathways/processes that are underpinning this quality trait.

Tools for the prediction of meat quality in the commercial setting are lacking at present. Measurement of pH at 45 minutes *post mortem* (pH _45_) has been applied to obtain an early indication of the final meat quality [72]. However, the general correlation between pH _45_ and WHC is not satisfactory as our study has shown a high level of variation in drip loss across just 30 samples presenting similar pH _45_ values. Kauffman et al., [73] in a study with five different WHC phenotypes, produced a categorisation accuracy of 52% for pH _45_ and 57% for pH _45_ and colour. The proteins identified in this study may have potential to overcome some of these issues, subject to validation in a larger set of samples. Increasing the number of samples (Pooling IP and LDrip or IP and HDrip) improved accuracy, so using a high-throughput technique with a higher number of samples, accuracy would likely be further increased. In addition, the approach of mining the centrifugal drip proteome for biomarkers could be applied to the prediction of other key meat quality traits such as tenderness and fat content.

### *Post mortem* degradation in extreme WHC phenotypes

One way ANOVA (p ≤ 0.05) analyses were performed to reveal spots that were significantly different across the three days *post mortem* in the two extreme phenotypes (HDrip and LDrip, IP having been analysed in [2]. Seventy-nine and 72 spots significantly changed (p ≤ 0.05) in abundance across days *post mortem* in HDrip and LDrip phenotypes respectively, whereas a considerably higher amount of spots (136) was found to be altered in IP [2]. This might indicate a reduction in soluble proteins appearing in centrifugal drip in extreme phenotypes.

Twenty of the 79 spots that were identified to be significantly changing across days *post mortem* in HDrip phenotype were characterised by mass spectrometry, and 52 proteins/peptides were identified (Table 6A and S2 Table). Thirty-three proteins/peptides were identified in 13 of the 72 spots/proteins changing across days *post mortem* in LDrip phenotype (Table 6B and S2 Table). Several proteins/peptides were identified in more than one spot. Fig 3A of our previous study Di Luca *et al*., [15] and S1 Fig shows the location of the spots characterised by mass spectrometric analysis. A number of spots/proteins which fluctuate over the ageing period were common between phenotypes [34 spots between HDrip and LDrip, 52 spots between HDrip and IP, 51 spots between LDrip and IP, 30 spots common between all three phenotypes (HDrip, LDrip and IP), the IP was analysed in Di Luca *et al*., [2]], suggesting meat ageing is not greatly affected by these drip loss phenotypes.

**Table 6:** ANOVA p value, fold changes (calculated from the mean normalised volumes between the groups that shows the maximum of the changes) and average normalised spot volumes of the 20 (A) and 13 (B) spots characterised by mass spectrometry in the *post mortem* comparisons respectively in the HDrip (A) and LDrip (B) phenotype.

**Spot**^**a**^	**ANOVA (p)**	**Fold change**	**(A) Mean normalised volume in the HDrip phenotype**		
			**HDrip Day 1**	**HDrip Day 3**	**HDrip Day 7**
1287	0.043	5.1	0.992^a^^b^	5.056^a^^b^	1.111^a^^b^
103	0.013	3.7	1.877^a^^@^	0.511^b^^@^	1.236^a^^b^
35	0.011	3	0.314^a^	0.568^a^	0.934^b^
227	0.021	2.7	0.646^a^	0.928^a^^b^	1.732^b^
329	0.03	2.6	0.687^a^	1.766^b^^@^	0.917^a^^b^
321	0.012	2.3	0.795^a^	0.413^b^	0.932^a^
27	0.015	2.2	0.705^a^	0.783^a^	1.574^b^
147	4.48E-04	2.1	0.422^a^	0.641^b^	0.881^c^
47	6.41E-04	1.8	0.65^a^	1.176^b^	1.188^b^
1360	0.007	1.7	1.683^a^^@^	1.324^b^	1.01^b^
1050	0.017	1.7	0.992^a^	0.888^a^	1.479^b^
1076	0.008	1.6	1.721^a^	1.307^b^	1.058^b^
68	0.011	1.5	1.402^a^	1.223^a^^b^	0.941^b^
857	0.017	1.5	1.625^a^	1.305^b^	1.099^b^
99	0.042	1.5	1.512^a^	1.023^b^^@^	1.037^b^^@^
1078	0.032	1.4	1.533^a^	1.256^a^^b^	1.077^b^
807	0.013	1.3	1.022^a^	0.979^a^	1.31^b^
1264	0.045	1.3	1.081^a^^b^	1.276^a^	0.969^b^
1000	0.013	1.3	1.512^a^	1.264^a^^b^	1.159^b^
65	0.001	1.2	1.276^a^	1.05^b^	1.048^b^
**Spot**^a^	**ANOVA (p)**	**Fold change**	**(B) Mean normalised volume in the LDrip phenotype**		
			**LDrip Day 1**	**LDrip Day 3**	**LDrip Day 7**
12	0.006	3.1	0.493^a^	0.82^a^^b^	1.512^b^
106	0.016	2.9	0.504^a^	0.803^a^^b^	1.442^b^
305	0.001	2.3	0.691^a^	1.32^a^^b^	1.594^b^
1287	0.05	2.3	0.973^a^^b^	1.984^a^^b^	0.86^a^^b^
280	0.009	2.1	0.99^a^	1.897^a^^b^	2.05^b^
498	0.000682	2	1.743^a^	1.0380^b^	0.887^b^
100	0.037	1.7	1.668^a^	0.970^b^	1.103^b^
21	0.012	1.7	1.591^a^	1.399^a^^b^	0.928^b^
1290	8.594e-004	1.5	0.898^a^	1.162^b^	1.324^b^
1192	0.003	1.5	1.083^a^	1.120^a^	1.586^b^
54	0.021	1.4	1.52^a^	1.069^b^	1.20^b^
1279	0.018	1.3	1.396^a^	1.054^b^	1.069^b^
591	0.049	1.2	1.42^a^^b^	1.547^a^^b^	1.246^a^^b^

Within rows, different superscripts indicate significantly different means at the 5% level (following Tukey Kramer post hoc analysis).

^@^indicates that the p-value was below 0.1.

^a^Spot numbers refer to Fig 3 in [15].

In all these comparisons, as with the multivariate approaches, the proteins identified fall generally into the following classes, structural proteins (e.g. titin, tropomyosin), metabolic enzymes (e.g. triosephosphate isomerase, adenylate kinase), and stress response proteins (e.g. HSP70, DJ 1 protein). The differences in the spot abundance pattern across the days *post mortem* in the different phenotypes are noteworthy. Indeed, while structural proteins are generally increasing in abundance in all three phenotypes, the other classes of proteins follow an irregular pattern in the HDrip and LDrip phenotypes, whereas the spot abundance of the IP phenotype show energy metabolism proteins increase while stress related proteins decrease. Few glycolytic enzymes were identified in both HDrip and LDrip comparisons.

Structural proteins were observed to increase in abundance across the ageing period, but there was no major effect of phenotype on this change. The calpain system plays a role in the degradation of many muscle proteins [74] and it is known that their activity is influenced by pH values of the *post mortem* muscle [74,75]. No differences were observed in pH or shear force across phenotypes (Table 1), which could explain the similarity in the abundance pattern of the structural proteins of the three phenotypes. The spot clustering in the PCA and in the hierarchical clustergram show a similar pattern across the ageing timepoint in all three phenotypes, which is especially evident at day 7 *post mortem*. Interestingly there are similarities also with the ANOVA analysis. Indeed, structural proteins were the more abundant at day 7 *post mortem* together to energy metabolism proteins, whereas stress related, transport and again energy metabolism proteins where more abundant at day 1 and 3 (irregular pattern observed in ANOVA). It is of interest to note that a relatively small proportion (~25%) of the spots seem to be fragments of the parent proteins.

Few stress related proteins were identified to change in abundance over time in the HDrip and LDrip time courses, and those identified showed an irregular pattern across the days *post mortem*. Previous work focusing on *post mortem* changes in the IP only [2] showed a high number of stress related proteins were identified at day 1, which decreased in abundance across the ageing period. Interestingly, HSPs were also more abundant in the IP compared to HDrip and LDrip at day 1 *post mortem* [15]. These proteins are essential for maintaining normal cellular activity because they associate with misfolded proteins, preventing their aggregation into large deleterious complexes. Moreover, HSP proteins enable cells to overcome stressful conditions by aiding the renaturation process of misfolded proteins [76]. A reduced abundance of these proteins in the extreme phenotypes for WHC may allude to a wider association of HSPs to the intermediate phenotype for drip loss and may reflect the heat shock response to cellular stress.

## Conclusion

This study investigated the *post mortem* processes associated with ageing in pork of divergent water holding capacity and sought to identify novel biomarkers of drip loss. Proteomic profiles over the *post mortem* ageing period were not strongly influenced by WHC phenotype. However, proteomic biomarkers associated with particular WHC phenotypes were identified using 2-D DIGE technology coupled with l1-regularized logistic regression. Our studies suggest that l1-regularized logistic regression is a promising approach to the identification of protein biomarkers for meat quality traits. Using this method, a high level of specificity (72–73%) was observed for some comparisons, showing that this approach has enhanced our understanding of this phenotype and has the capacity to provide new biomarker panels for early meat quality prediction.

## Supporting Information

S1 Fig Representative 2-D DIGE gel images which show the 22 new spots identified by PEAKS Studio 6. Centrifugal drip proteins were separated by 2-D DIGE using immobilised pH 4–7 gradients (24 cm, linear) in the first dimension and 12% SDS-PAGE in the second dimension. The gel image is from a CyDye3-labelled reference sample (pool of all samples used). This new data were merged together in the online proteome map for porcine exudate derived from 36 2-D DIGE gels presented in our previous study [15]. This database is available as part of the UCD-2DPAGE database under the tag ‘Porcine Database’ (http://proteomics-portal.ucd.ie).
(TIF)

S1 Table Identified protein/fragment spots in porcine centrifugal drip with Peaks Studio 6. ^a^Spot numbers refers to Fig 3 in our previous study [15]. ^b^Molecular weight of the protein.
(DOC)

S2 Table Biological function of the identified protein/fragment spots in porcine centrifugal drip (phenotypes and time course comparisons). ^a^Spot numbers refer to Fig 3 in [15]. ^b^Biological process of the proteins obtained using PANTHER analysis [25]. ^c^Comparison where the protein was significantly different [phenotype (PH), time course (TC)].
(DOC)
