# Supplementary figures and images for: Comparative Proteomic Profiling of Divergent Phenotypes for Water Holding Capacity across the Post Mortem Ageing Period in Porcine Muscle Exudate
Source: PLoS One. 2016 Mar 7;11(3):e0150605. doi: 10.1371/journal.pone.0150605 (PMC4780776; doi:10.1371/journal.pone.0150605)

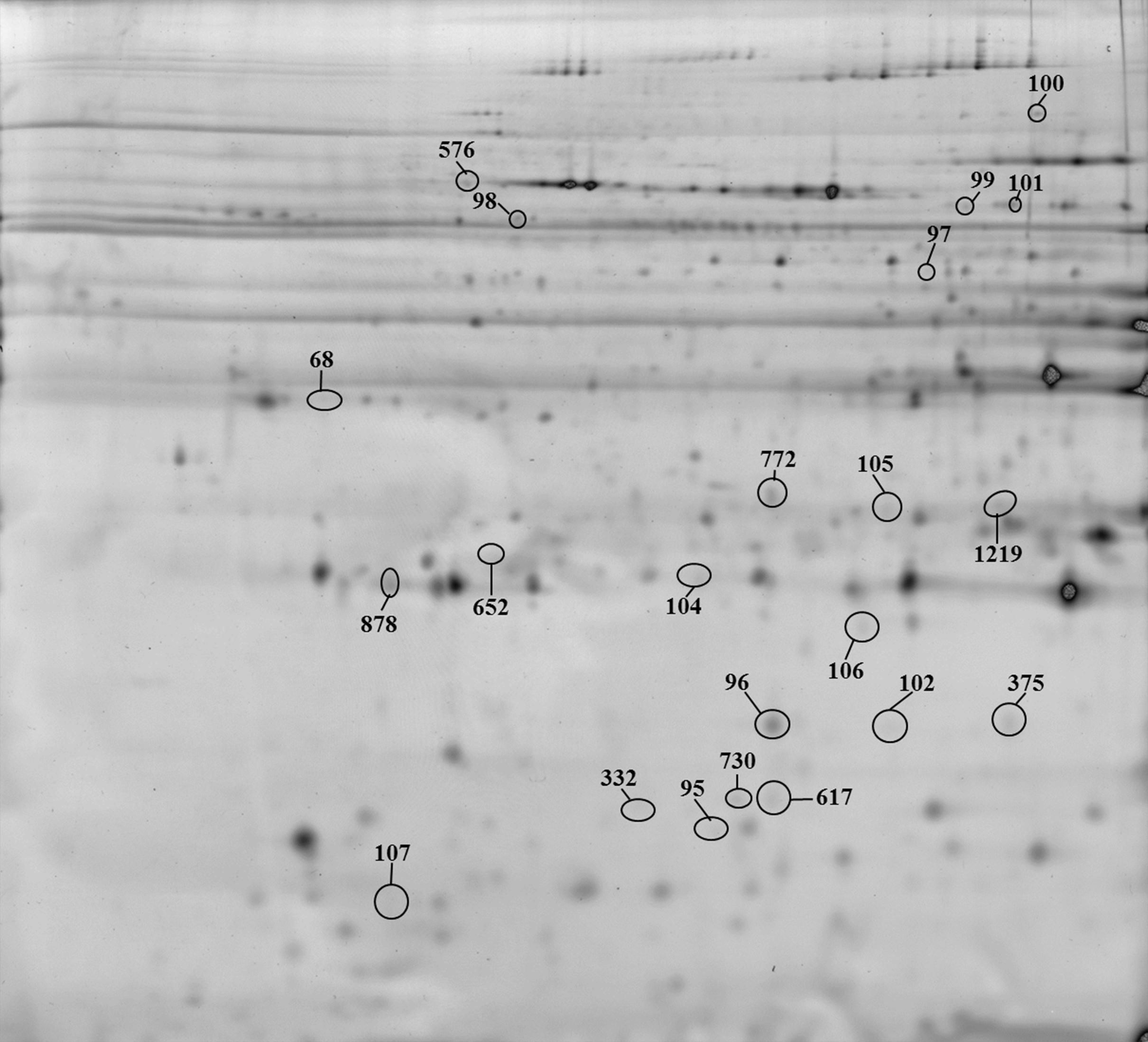

Supplement: S1 Fig — Centrifugal drip proteins were separated by 2-D DIGE using immobilised pH 4–7 gradients (24 cm, linear) in the first dimension and 12% SDS-PAGE in the second dimension. The gel image is from a CyDye3-labelled reference sample (pool of all samples used). This new data were merged together in the online proteome map for porcine exudate derived from 36 2-D DIGE gels presented in our previous study [15]. This database is available as part of the UCD-2DPAGE database under the tag ‘Porcine Database’ (http://proteomics-portal.ucd.ie). (TIF) [file pone.0150605.s001.tif]
