# Supplementary material for: Comparative Proteomic Profiling of Divergent Phenotypes for Water Holding Capacity across the Post Mortem Ageing Period in Porcine Muscle Exudate
Source: PLoS One. 2016 Mar 7;11(3):e0150605. doi: 10.1371/journal.pone.0150605 (PMC4780776; doi:10.1371/journal.pone.0150605)
Supplement: S1 Table — aSpot numbers refers to Fig 3 in our previous study [15]. bMolecular weight of the protein. (DOC) [file pone.0150605.s002.doc]

**S1 Table. Identified protein/fragment spots in porcine centrifugal drip with Peaks Studio 6.**

| **Spot^a^** | **Accession** | **Coverage** | | **Peptides** | **MW (kDa)^b^** | **Identification** |
| --- | --- | --- | --- | --- | --- | --- |
| **12** | P42639|TPM1_PIG | 37 | | 14 | 32.7 | Tropomyosin alpha-1 chain |
| **12** | P67937|TPM4_PIG | 35 | | 12 | 28.5 | Tropomyosin alpha-4 chain |
| **12** | A1XQV4|TPM3_PIG | 25 | | 12 | 33.01 | Tropomyosin alpha-3 chain |
| **27** | Q5XLD3|KCRM_PIG | 29 | | 9 | 43.1 | Creatine kinase M-type |
| **27** | P09571|TRFE_PIG | 11 | | 6 | 77 | Serotransferrin |
| **47** | Q1KYT0|ENOB_PIG | 19 | | 6 | 47.1 | Beta-enolase |
| **47** | Q7SIB7|PGK1_PIG | 15 | | 5 | 44.6 | Phosphoglycerate kinase 1 |
| **53** | P08835|ALBU_PIG | 20 | | 16 | 69.7 | Serum albumin |
| **57** | P42639|TPM1_PIG | 57 | | 23 | 32.7 | Tropomyosin alpha-1 chain |
| **57** | A1XQV4|TPM3_PIG | 24 | | 10 | 33.1 | Tropomyosin alpha-3 chain |
| **57** | P00339|LDHA_PIG | 19 | | 6 | 36.6 | L-lactate dehydrogenase A chain |
| **57** | P67937|TPM4_PIG | 19 | | 7 | 28.5 | Tropomyosin alpha-4 chain |
| **57** | A5A8V7|HS71L_PIG | 6 | | 4 | 70.3 | Heat shock 70 kDa protein 1-like |
| **57** | Q6S4N2|HS71B_PIG | 4 | | 3 | 70.1 | Heat shock 70 kDa protein 1B |
| **57** | P34930|HS71A_PIG | 4 | | 3 | 70.1 | Heat shock 70 kDa protein 1A |
| **65** | O02697|PK3CG_PIG | 6 | | 7 | 126.7 | Phosphatidylinositol 4,5-bisphosphate 3-kinase catalytic subunit gamma isoform |
| **66** | Q5XLD3|KCRM_PIG | 3 | | 2 | 43.1 | Creatine kinase M-type |
| **68** | P50828|HEMO_PIG | 5 | | 2 | 51.3 | Hemopexin |
| **90** | P52552|PRDX2_PIG | 58 | | 11 | 14.2 | Peroxiredoxin-2 (Fragment) |
| **91** | A5A8V7|HS71L_PIG | 14 | | 8 | 70.3 | Heat shock 70 kDa protein 1-like |
| **91** | Q04967|HSP76_PIG | 9 | | 7 | 71.1 | Heat shock 70 kDa protein 6 |
| **91** | P34930|HS71A_PIG | 9 | | 6 | 70.1 | Heat shock 70 kDa protein 1A |
| **92** | Q6S4N2|HS71B_PIG | 18 | | 10 | 70.1 | Heat shock 70 kDa protein 1B |
| **92** | Q04967|HSP76_PIG | 14 | | 7 | 71.1 | Heat shock 70 kDa protein 6 |
| **92** | A5A8V7|HS71L_PIG | 8 | | 4 | 70.3 | Heat shock 70 kDa protein 1-like |
| **92** | P34934|HSP7X_PIG | 8 | | 3 | 42.3 | Heat shock 70 kDa protein (Fragment) |
| **93** | P50828|HEMO_PIG | 25 | | 11 | 51.3 | Hemopexin |
| **93** | P08835|ALBU_PIG | 7 | | 4 | 69.7 | Serum albumin |
| **95** | O46658|CP2DP_PIG | 2 | | 2 | 56.5 | Vitamin D(3) 25-hydroxylase |
| **96** | Q5G6V9|COF2_PIG | 42 | | 9 | 18.7 | Cofilin-2 |
| **96** | P10668|COF1_PIG | 21 | | 4 | 18.5 | Cofilin-1 |
| **97** | Q6PQZ2|CFTR_PIG | 1 | | 2 | 168.2 | Cystic fibrosis transmembrane conductance regulator |
| **98** | P50447|A1AT_PIG | 24 | | 12 | 47.2 | Alpha-1-antitrypsin |
| **99** | P29700|FETUA_PIG | 8 | | 4 | 38.4 | Alpha-2-HS-glycoprotein (Fragment) |
| **100** | Q6S4N2|HS71B_PIG | 10 | | 6 | 70.1 | Heat shock 70 kDa protein 1B |
| **100** | A5A8V7|HS71L_PIG | 8 | | 5 | 70.3 | Heat shock 70 kDa protein 1-like |
| **100** | Q04967|HSP76_PIG | 4 | | 2 | 71.1 | Heat shock 70 kDa protein 6 |
| **100** | Q03710|CFAB_PIG | 17 | | 4 | 16.8 | Complement factor B (Fragment) |
| **101** | Q1KYT0|ENOB_PIG | 7 | | 4 | 47.1 | Beta-enolase |
| **102** | Q5G6V9|COF2_PIG | 55 | | 11 | 18.7 | Cofilin-2 |
| **103** | Q2XQV4|ALDH2_PIG | 25 | | 15 | 56.9 | Aldehyde dehydrogenase, mitochondrial |
| **104** | P00571|KAD1_PIG | 33 | | 8 | 21.6 | Adenylate kinase isoenzyme 1 |
| **105** | A1XQU1|PSB7_PIG | 25 | | 9 | 30 | Proteasome subunit beta type-7 |
| **105** | Q1KYT0|ENOB_PIG | 4 | | 2 | 47.1 | Beta-enolase |
| **105** | Q5S1S4|CAH3_PIG | 8 | | 2 | 29.4 | Carbonic anhydrase 3 |
| **106** | P00571|KAD1_PIG | 24 | | 6 | 21.6 | Adenylate kinase isoenzyme 1 |
| **107** | Q9TV62|MYH4_PIG | 2 | | 4 | 223.2 | Myosin-4 |
| **107** | Q9TV61|MYH1_PIG | 2 | | 4 | 223.2 | Myosin-1 |
| **107** | Q9TV63|MYH2_PIG | 2 | | 3 | 223.1 | Myosin-2 |
| **116** | Q29371|TPIS_PIG | 20 | | 5 | 26.7 | Triosephosphate isomerase |
| **119** | Q1KYT0|ENOB_PIG | 36 | | 18 | 47.1 | Beta-enolase |
| **124** | A5GFS8|VAPB_PIG | 19 | | 4 | 27 | Vesicle-associated membrane protein-associated protein B |
| **153** | Q1KYT0|ENOB_PIG | 13 | | 5 | 47.1 | Beta-enolase |
| **153** | P80276|ALDR_PIG | 12 | | 5 | 35.9 | Aldose reductase |
| **170** | P08835|ALBU_PIG | 26 | | 15 | 69.5 | serum albumin |
| **170** | P08059|G6PI_PIG | 11 | | 5 | 63.1 | glucose-6-phosphate isomerase |
| **177** | P26234|VINC_PIG | 13 | | 13 | 123.9 | Vinculin |
| **177** | Q6S4N2|HS71B_PIG | 9 | | 5 | 70.1 | Heat shock 70 kDa protein 1B |
| **177** | P34930|HS71A_PIG | 9 | | 5 | 70.1 | Heat shock 70 kDa protein 1A |
| **177** | Q04967|HSP76_PIG | 7 | | 4 | 71.1 | Heat shock 70 kDa protein 6 |
| **177** | A5A8V7|HS71L_PIG | 7 | | 4 | 70.3 | Heat shock 70 kDa protein 1-like |
| **218** | P08835|ALBU_PIG | 69 | | 52 | 69.7 | serum albumin |
| **218** | P50828|HEMO_PIG | 40 | | 29 | 51.3 | hemopexin |
| **218** | Q9TV61|MYH1_PIG | 1 | | 2 | 223.2 | myosin-1 |
| **218** | Q9TV63|MYH2_PIG | 1 | | 2 | 223.2 | myosin-2 |
| **218** | P79293|MYH7_PIG | 1 | | 2 | 223.3 | myosin-7 |
| **239** | P50828|HEMO_PIG | 37 | | 20 | 51.3 | Hemopexin |
| **239** | Q5D891|PK3C3_PIG | 2 | | 2 | 101.2 | Phosphatidylinositol 3-kinase catalytic subunit type 3 |
| **277** | P08835|ALBU_PIG | 30 | | 19 | 69.7 | serum albumin |
| **277** | P50828|HEMO_PIG | 31 | | 14 | 51.3 | hemopexin |
| **277** | P50390|TTHY_PIG | 15 | | 2 | 16.1 | transthyretin |
| **305** | P00571|KAD1_PIG | 41 | | 10 | 21.6 | Adenylate kinase isoenzyme 1 |
| **321** | O77591|IMPA1_PIG | 38 | | 13 | 30.1 | Inositol monophosphatase 1 |
| **321** | Q5D891|PK3C3_PIG | 2 | | 2 | 101.2 | Phosphatidylinositol 3-kinase catalytic subunit type 3 |
| **321** | O77591|IMPA1_PIG | 33 | | 13 | 30.1 | Inositol monophosphatase 1 |
| **329** | Q06AB3|UCHL3_PIG | 45 | | 8 | 26.1 | Ubiquitin carboxyl-terminal hydrolase isozyme L3 |
| **332** | Q29371|TPIS_PIG | 10 | | 2 | 26.7 | Triosephosphate isomerase |
| **358** | Q06AB3|UCHL3_PIG | 33 | | 7 | 26.1 | Ubiquitin carboxyl-terminal hydrolase isozyme L3 |
| **375** | A5GFS8|VAPB_PIG | 10 | | 4 | 27 | Vesicle-associated membrane protein-associated protein B |
| **375** | P34930|HS71A_PIG | 10 | | 5 | 70.1 | Heat shock 70 kDa protein 1A |
| **375** | A5A8V7|HS71L_PIG | 6 | | 3 | 70.3 | Heat shock 70 kDa protein 1-like |
| **375** | Q04967|HSP76_PIG | 8 | | 3 | 71.1 | Heat shock 70 kDa protein 6 |
| **452** | P26234|VINC_PIG | 23 | | 29 | 123.9 | Vinculin |
| **503** | P50828|HEMO_PIG | 17 | | 7 | 51.3 | hemopexin |
| **503** | P08835|ALBU_PIG | 7 | | 4 | 69.7 | serum albumin |
| **531** | Q9N1F5|GSTO1_PIG | 27 | | 6 | 27.4 | Glutathione S-transferase omega-1 |
| **531** | Q29371|TPIS_PIG | 10 | | 3 | 26.7 | Triosephosphate isomerase |
| **566** | A5A8V7|HS71L_PIG | 24 | | 18 | 70.3 | Heat shock 70 kDa protein 1-like |
| **566** | Q6S4N2|HS71B_PIG | 25 | | 19 | 70.1 | Heat shock 70 kDa protein 1B |
| **566** | P34930|HS71A_PIG | 25 | | 19 | 70.1 | Heat shock 70 kDa protein 1A |
| **566** | Q04967|HSP76_PIG | 23 | | 18 | 71.1 | Heat shock 70 kDa protein 6 |
| **566** | P08835|ALBU_PIG | 4 | | 2 | 69.7 | Serum albumin |
| **576** | P52552|PRDX2_PIG | 14 | | 2 | 14.2 | Peroxiredoxin-2 (Fragment) |
| **617** | Q2EN76|NDKB_PIG | 32 | | 4 | 17.2 | Nucleoside diphosphate kinase B |
| **617** | P52552|PRDX2_PIG | 31 | | 4 | 14.2 | Peroxiredoxin-2 (Fragment) |
| **617** | Q29387|EF1G_PIG | 7 | | 3 | 49.6 | Elongation factor 1-gamma (Fragment) |
| **648** | Q7SIB7|PGK1_PIG | 34 | | 14 | 44.6 | Phosphoglycerate kinase 1 |
| **648** | Q6RI85|PGK2_PIG | 10 | | 3 | 44.9 | Phosphoglycerate kinase 2 |
| **652** | P08835|ALBU_PIG | 16 | | 12 | 69.7 | Serum albumin |
| **652** | Q8MJ76|FETA_PIG | 2 | | 2 | 68.6 | Alpha-fetoprotein |
| **652** | P08835|ALBU_PIG | 6 | | 4 | 69.7 | Serum albumin |
| **652** | P11708|MDHC_PIG | 36 | | 10 | 36.4 | Malate dehydrogenase, cytoplasmic |
| **730** | Q29371|TPIS_PIG | 26 | | 5 | 26.7 | Triosephosphate isomerase |
| **772** | A1XQU1|PSB7_PIG | 27 | | 9 | 30 | Proteasome subunit beta type-7 |
| **807** | P34930|HS71A_PIG | 28 | | 24 | 70.1 | Heat shock 70 kDa protein 1A |
| **807** | A5A8V7|HS71L_PIG | 31 | | 21 | 70.3 | Heat shock 70 kDa protein 1-like |
| **807** | Q04967|HSP76_PIG | 22 | | 17 | 71.1 | Heat shock 70 kDa protein 6 |
| **807** | P34934|HSP7X_PIG | 11 | | 7 | 42.1 | Heat shock 70 kDa protein (Fragment) |
| **807** | Q6S4N2|HS71B_PIG | 50 | | 39 | 70.1 | Heat shock 70 kDa protein 1B |
| **857** | P29700|FETUA_PIG | 22 | | 8 | 38.4 | Alpha-2-HS-glycoprotein (Fragment) |
| **857** | Q6QAQ1|ACTB_PIG | 7 | | 2 | 41.7 | Actin, cytoplasmic 1 |
| **878** | P08835|ALBU_PIG | 9 | | 5 | 69.7 | serum albumin |
| **878** | P52552|PRDX2_PIG | 16 | | 2 | 14.2 | peroxiredoxin-2 (fragment) |
| **878** | P50828|HEMO_PIG | 8 | | 2 | 51.3 | hemopexin |
| **935** | P08835|ALBU_PIG | 26 | | 17 | 68.7 | Serum albumin |
| **962** | P00339|LDHA_PIG | 35 | | 11 | 36.6 | L-lactate dehydrogenase A chain |
| **962** | Q9TSX5|LDHC_PIG | 6 | | 2 | 36 | L-lactate dehydrogenase C chain |
| **999** | P11607|AT2A2_PIG | 2 | | 3 | 114.8 | Sarcoplasmic/endoplasmic reticulum calcium ATPase 2 |
| **1000** | P42639|TPM1_PIG | 33 | | 16 | 32.7 | Tropomyosin alpha-1 chain |
| **1000** | P67937|TPM4_PIG | 38 | | 15 | 28.5 | Tropomyosin alpha-4 chain |
| **1000** | A1XQV4|TPM3_PIG | 26 | | 12 | 33.1 | Tropomyosin alpha-3 chain |
| **1007** | P00339|LDHA_PIG | 36 | | 12 | 36.6 | L-lactate dehydrogenase A chain |
| **1007** | P03974|TERA_PIG | 7 | | 6 | 89.3 | Transitional endoplasmic reticulum ATPase |
| **1007** | Q9TSX5|LDHC_PIG | 6 | | 2 | 36 | L-lactate dehydrogenase C chain |
| **1007** | P00336|LDHB_PIG | 5 | | 2 | 36.6 | L-lactate dehydrogenase B chain |
| **1011** | P08835|ALBU_PIG | 32 | | 24 | 69.7 | Serum albumin |
| **1011** | Q29558|MAOX_PIG | 11 | | 7 | 62 | NADP-dependent malic enzyme (Fragment) |
| **1011** | Q8MJ76|FETA_PIG | 4 | | 3 | 68.6 | Alpha-fetoprotein |
| **1050** | A5A8V7|HS71L_PIG | 7 | | 5 | 70.3 | Heat shock 70 kDa protein 1-like |
| **1050** | Q6S4N2|HS71B_PIG | 5 | | 4 | 70.1 | Heat shock 70 kDa protein 1B |
| **1050** | P34930|HS71A_PIG | 5 | 4 | | 70.1 | Heat shock 70 kDa protein 1A |
| **1050** | A1XQV4|TPM3_PIG | 8 | 2 | | 33.1 | Tropomyosin alpha-3 chain |
| **1061** | Q6QAQ1|ACTB_PIG | 10 | 2 | | 41.7 | Actin, cytoplasmic 1 |
| **1061** | P68137|ACTS_PIG | 10 | 2 | | 42 | Actin, alpha skeletal muscle |
| **1076** | P29700|FETUA_PIG | 22 | | 8 | 38.4 | Alpha-2-HS-glycoprotein (Fragment) |
| **1078** | Q1KYT0|ENOB_PIG | 31 | | 15 | 47.1 | Beta-enolase |
| **1078** | P00339|LDHA_PIG | 32 | | 11 | 36.6 | L-lactate dehydrogenase A chain |
| **1078** | P11708|MDHC_PIG | 19 | | 6 | 36.4 | Malate dehydrogenase, cytoplasmic |
| **1078** | P80276|ALDR_PIG | 22 | | 9 | 35.9 | Aldose reductase |
| **1078** | P00336|LDHB_PIG | 8 | | 3 | 36.6 | L-lactate dehydrogenase B chain |
| **1078** | Q9TSX5|LDHC_PIG | 6 | | 2 | 36 | L-lactate dehydrogenase C chain |
| **1078** | Q1KYT0|ENOB_PIG | 25 | | 10 | 47.1 | Beta-enolase |
| **1078** | B1PK17|ACY2_PIG | 35 | | 11 | 35.6 | Aspartoacylase |
| **1078** | P00636|F16P1_PIG | 5 | | 2 | 36.8 | Fructose-1,6-bisphosphatase 1 |
| **1100** | Q29371|TPIS_PIG | 20 | | 5 | 26.7 | Triosephosphate isomerase |
| **1100** | Q1KYT0|ENOB_PIG | 12 | | 7 | 47.1 | Beta-enolase |
| **1135** | Q1KYT0|ENOB_PIG | 7 | | 2 | 47.1 | Beta-enolase |
| **1192** | Q6S4N2|HS71B_PIG | 57 | | 37 | 70.1 | Heat shock 70 kDa protein 1B |
| **1192** | P34930|HS71A_PIG | 45 | | 30 | 70.1 | Heat shock 70 kDa protein 1A |
| **1192** | P08835|ALBU_PIG | 55 | | 33 | 69.7 | Serum albumin |
| **1192** | Q04967|HSP76_PIG | 23 | | 16 | 71.1 | Heat shock 70 kDa protein 6 |
| **1192** | A5A8V7|HS71L_PIG | 28 | | 17 | 70.3 | Heat shock 70 kDa protein 1-like |
| **1219** | Q29371|TPIS_PIG | 39 | | 7 | 26.7 | Triosephosphate isomerase |
| **1219** | P08835|ALBU_PIG | 16 | | 10 | 69.7 | Serum albumin |
| **1219** | Q5XLD3|KCRM_PIG | 20 | | 7 | 43.1 | Creatine kinase M-type |
| **1264** | Q6S4N2|HS71B_PIG | 15 | | 9 | 70.1 | Heat shock 70 kDa protein 1B |
| **1264** | P08835|ALBU_PIG | 14 | | 10 | 69.7 | Serum albumin |
| **1264** | Q04967|HSP76_PIG | 5 | | 3 | 71.1 | Heat shock 70 kDa protein 6 |
| **1264** | A5A8V7|HS71L_PIG | 6 | | 3 | 70.3 | Heat shock 70 kDa protein 1-like |
| **1270** | P61288|TCTP_PIG | 15 | | 2 | 19.6 | Translationally-controlled tumor protein |
| **1279** | P01846|LAC_PIG | 34 | | 2 | 11 | Ig lambda chain C region |
| **1290** | Q6S4N2|HS71B_PIG | 44 | | 24 | 70.1 | Heat shock 70 kDa protein 1B |
| **1290** | Q04967|HSP76_PIG | 20 | | 13 | 71.1 | Heat shock 70 kDa protein 6 |
| **1290** | A5A8V7|HS71L_PIG | 18 | | 10 | 70.3 | Heat shock 70 kDa protein 1-like |
| **1290** | P08835|ALBU_PIG | 16 | | 8 | 69.7 | Serum albumin |
| **1290** | P08059|G6PI_PIG | 9 | | 5 | 63.1 | Glucose-6-phosphate isomerase |
| **1291** | P08835|ALBU_PIG | 59 | | 42 | 69.7 | Serum albumin |
| **1291** | P50828|HEMO_PIG | 10 | | 6 | 51.3 | Hemopexin |
| **1291** | Q8MJ76|FETA_PIG | 3 | | 2 | 68.6 | Alpha-fetoprotein |
| **1333** | P28839|AMPL_PIG | 75 | | 3 | 4 | Cytosol aminopeptidase (Fragment) |
| **1360** | P29700|FETUA_PIG | 37 | | 13 | 38.4 | Alpha-2-HS-glycoprotein (Fragment) |
|  |  |  |  | |  |  |

^a^Spot numbers refers to Fig 3 in our previous study [15]. ^b^Molecular weight of the protein.
