# Supplementary material for: Comparative Proteomic Profiling of Divergent Phenotypes for Water Holding Capacity across the Post Mortem Ageing Period in Porcine Muscle Exudate
Source: PLoS One. 2016 Mar 7;11(3):e0150605. doi: 10.1371/journal.pone.0150605 (PMC4780776; doi:10.1371/journal.pone.0150605)
Supplement: S2 Table — aSpot numbers refer to Fig 3 in [15]. bBiological process of the proteins obtained using PANTHER analysis [25]. cComparison where the protein was significantly different [phenotype (PH), time course (TC)]. (DOC) [file pone.0150605.s003.doc]

**S2 Table. Biological function of the identified protein/fragment spots in porcine centrifugal drip (phenotypes and time course comparisons).**

| **Spot^a^** | **Protein identified** | **Gene name** | **Biological process ^b^** | **Changing in phenotype/time course comparison^c^** |
| --- | --- | --- | --- | --- |
| **12** | Beta-tropomyosin | TPM2 | muscle contraction; cell motion; cellular component morphogenesis | TC - LDrip |
| **12** | Tropomyosin alpha-1 chain | TPM1 | muscle contraction; cell motion; cellular component morphogenesis | TC - LDrip |
| **12** | Tropomyosin alpha-4 chain | TPM4 | muscle contraction; cell motion; cellular component morphogenesis | TC - LDrip |
| **12** | Tropomyosin alpha-3 chain | TPM3 | muscle contraction; cell motion; cellular component morphogenesis | TC - LDrip |
| **21** | Annexin A7 | ANXA7 | intracellular protein transport; signal transduction; lipid metabolic process; cell motion; signal transduction | TC - LDrip |
| **27** | Creatine kinase M-type | **CKM** | muscle contraction; metabolic process | TC – HDrip |
| **27** | Serotransferrin | **TF** | macrophage activation; induction of apoptosis; cell surface receptor linked signal transduction; intracellular signaling cascade; cell-cell signalling; signal transduction | TC – HDrip |
| **35** | Titin | TTN | assemblage and functioning of vertebrate striated muscles | TC – HDrip |
| **47** | Phosphoglycerate kinase 1 | PGK1 | carbohydrate metabolic process | TC – HDrip |
| **47** | Beta-enolase | ENO3 | glycolysis | TC – HDrip |
| **54** | Serum albumin | ALB | transport | TC - LDrip |
| **54** | Transferrin | TF | macrophage activation; induction of apoptosis; cell surface receptor linked signal transduction; intracellular signaling cascade; cell-cell signalling; signal transduction | TC - LDrip |
| **65** | Filaggrin-2 | FLG2 | protein metabolic process; cellular component morphogenesis; ectoderm development | TC – HDrip |
| **65** | Phosphatidylinositol 4,5-bisphosphate 3-kinase catalytic subunit gamma isoform | PIK3CG | protein targeting; endocytosis; negative regulation of apoptosis; transmembrane receptor protein tyrosine kinase signaling pathway; phosphate metabolic process  phospholipid metabolic process | TC – HDrip |
| **68** | Hemopexin | HPX | vitamin transport | TC – HDrip |
| **99** | Alpha-2-HS-glycoprotein (Fragment) | AHSG | immune system process; protein metabolic process; mesoderm development; skeletal system development | TC – HDrip |
| **100** | Heat shock 70 kDa protein 1B | HSPA1B | immune system process; protein metabolic process; response to stress | TC - LDrip |
| **100** | Heat shock 70 kDa protein 1-like | HSPA1L | immune system process; protein metabolic process; response to stress | TC - LDrip |
| **100** | Heat shock 70 kDa protein 6 | HSPA6 | immune system process; protein metabolic process; response to stress | TC - LDrip |
| **100** | Complement factor B (Fragment) | CFB | complement activation; signal transduction; cell-cell adhesion; proteolysis; blood coagulation | TC - LDrip |
| **101** | Beta-enolase | ENO3 | glycolysis | PH day 3 |
| **103** | Aldehyde dehydrogenase, mitochondrial | ALDH2 | carbohydrate, cellular amino acid and derivative metabolic process | TC – HDrip |
| **106** | Adenylate kinase isoenzyme 1 | AK1 | nucleobase, nucleoside, nucleotide and nucleic acid metabolic process | TC - LDrip |
| **109** | Stress-induced-phosphoprotein 1 | STIP1 | immune system process; protein metabolic process; response to stress | PH day 3; PH day 7 |
| **116** | Triosephosphate isomerase (Fragment) | TPI | fatty acid biosynthesis; gluconeogenesis; glycolysis | PH day 7 |
| **116** | Phosphoglucomutase 1 (Fragment) | PGM1 | carbohydrate metabolic process | PH day 7 |
| **147** | Titin | TTN | assemblage and functioning of vertebrate striated muscles | TC – HDrip |
| **176** | Vimentin | VIM | ectoderm development; cellular component morphogenesis | PH day 3 |
| **177** | Heat shock 70 kDa protein 1A | HSPA1A | immune system process; protein metabolic process; response to stress | PH day 7 |
| **177** | Vinculin | VCL | cell adhesion; cell motion; intracellular signaling cascade; cellular component morphogenesis | PH day 7 |
| **177** | Heat shock 70 kDa protein 1B | HSPA1B | immune system process; protein metabolic process; response to stress | PH day 7 |
| **177** | Heat shock 70 kDa protein 6 | HSPA6 | immune system process; protein metabolic process; response to stress | PH day 7 |
| **177** | Heat shock 70 kDa protein 1-like | HSPA1L | immune system process; protein metabolic process; response to stress | PH day 7 |
| **227** | Triosephosphate isomerase | TPI1 | fatty acid biosynthesis; gluconeogenesis; glycolysis | TC – HDrip |
| **280** | Adenylate kinase isoenzyme 1 | AK1 | nucleobase, nucleoside, nucleotide and nucleic acid metabolic process | TC - LDrip |
| **305** | Protein DJ-1 | PARK7 | immune system process; nucleobase, nucleoside, nucleotide and nucleic acid metabolic process; protein metabolic process; response to stress | TC - LDrip |
| **305** | Adenylate kinase isoenzyme 1 | AK1 | nucleobase, nucleoside, nucleotide and nucleic acid metabolic process | TC - LDrip |
| **321** | Inositol monophosphatase 1 | IMPA1 | sulfur metabolic process; nucleobase, nucleoside, nucleotide and nucleic acid metabolic process; lipid metabolic process | TC – HDrip |
| **321** | Phosphatidylinositol 3-kinase catalytic subunit type 3 | PIK3C3 | protein targeting; endocytosis; negative regulation of apoptosis; transmembrane receptor protein tyrosine kinase signaling pathway; phosphate metabolic process; phospholipid metabolic process | TC – HDrip |
| **326** | Phosphoglucomutase 1 | PGM1 | carbohydrate metabolic process | PH day 7 |
| **329** | Ubiquitin carboxyl-terminal hydrolase isozyme L3 | UCHL3 | protein metabolic process | TC – HDrip |
| **498** | Apolipoprotein A-I | APOA1 | blood circulation; lipid transport; lipid metabolic process | TC - LDrip |
| **566** | Heat shock 70 kDa protein 1A | HSPA1A | immune system process; protein metabolic process; response to stress | PH day 7 |
| **566** | Heat shock 70 kDa protein 1-like | HSPA1L | immune system process; protein metabolic process; response to stress | PH day 7 |
| **566** | Heat shock protein 68 | Hsp68 | immune system process; protein metabolic process; response to stress | PH day 7 |
| **566** | Heat shock cognate 71 | HSPA8 | immune system process; protein metabolic process; response to stress | PH day 7 |
| **566** | Luminal-binding protein 2 | BIP2 | immune system process; protein metabolic process; response to stress | PH day 7 |
| **566** | Heat shock 70 kDa protein 1B | HSPA1B | immune system process; protein metabolic process; response to stress | PH day 7 |
| **566** | Heat shock 70 kDa protein 6 | HSPA6 | immune system process; protein metabolic process; response to stress | PH day 7 |
| **566** | Serum albumin | ALB | transport | PH day 7 |
| **591** | Serum albumin | ALB | transport | TC - LDrip |
| **591** | Adenosylhomocysteinase | AHCY | nucleobase, nucleoside, nucleotide and nucleic acid metabolic process | TC - LDrip |
| **652** | Serum albumin | ALB | transport | PH day 7 |
| **652** | Alpha-fetoprotein | AFP | transport | PH day 7 |
| **652** | Malate dehydrogenase, cytoplasmic | MDH1 | tricarboxylic acid cycle; carbohydrate metabolic process | PH day 7 |
| **807** | Heat shock 70kDa protein 1A | HSPA1A | immune system process; protein metabolic process; response to stress | TC – HDrip |
| **807** | Heat shock 70 kDa protein 1L | HSPA1L | immune system process; protein metabolic process; response to stress | TC – HDrip |
| **807** | Heat shock cognate 71 kDa protein | HSPA8 | immune system process; protein metabolic process; response to stress | TC – HDrip |
| **807** | Heat shock cognate 70 kDa protein 1 | HSPB | immune system process; protein metabolic process; response to stress | TC – HDrip |
| **807** | Heat shock protein 68 | HSP68 | immune system process; protein metabolic process; response to stress | TC – HDrip |
| **807** | Heat shock 70 kDa protein 6 | HSPA6 | immune system process; protein metabolic process; response to stress | TC – HDrip |
| **807** | Heat shock 70 kDa protein (Fragment) | HSP70 | immune system process; protein metabolic process; response to stress | TC – HDrip |
| **807** | Heat shock 70 kDa protein 1B | HSPA1B | immune system process; protein metabolic process; response to stress | TC – HDrip |
| **857** | Alpha-2-HS-glycoprotein (Fragment) | AHSG | immune system process; protein metabolic process; mesoderm development; skeletal system development | TC – HDrip |
| **857** | Actin, cytoplasmic 1 | ACTB | intracellular protein transport; exocytosis; endocytosis; mitosis; cytokinesis; cellular component morphogenesis | TC – HDrip |
| **935** | Albumin (Fragment) | ALB | transport | PH day 7 |
| **935** | Serum albumin | ALB | transport | PH day 7 |
| **1000** | Beta-tropomyosin | TPM2 | structural constituent of cytoskeleton; muscle contraction; cell motion; cellular component morphogenesis | TC – HDrip |
| **1000** | Tropomyosin alpha-1 chain | TPM1 | muscle contraction; cell motion; cellular component morphogenesis | TC – HDrip |
| **1000** | Tropomyosin alpha-4 chain | TPM4 | muscle contraction; cell motion; cellular component morphogenesis | TC – HDrip |
| **1000** | Tropomyosin alpha-3 chain | TPM3 | muscle contraction; cell motion; cellular component morphogenesis | TC – HDrip |
| **1050** | Tropomyosin alpha-1 chain | TPM1 | muscle contraction; cell motion; cellular component morphogenesis | TC – HDrip |
| **1050** | Heat shock 70 kDa protein 1-like | HSPA1L | immune system process; protein metabolic process; response to stress | TC – HDrip |
| **1050** | Heat shock 70 kDa protein 1B | HSPA1B | immune system process; protein metabolic process; response to stress | TC – HDrip |
| **1050** | Heat shock 70 kDa protein 1A | HSPA1A | immune system process; protein metabolic process; response to stress | TC – HDrip |
| **1050** | Tropomyosin alpha-3 chain | TPM3 | muscle contraction; cell motion; cellular component morphogenesis | TC – HDrip |
| **1061** | Vimentin | VIM | ectoderm development; cellular component morphogenesis | PH day 7 |
| **1061** | Filamin-A | FLNA | protein localization at cell surface | PH day 7 |
| **1061** | Actin, cytoplasmic 1 | ACTB | intracellular protein transport; exocytosis; endocytosis; mitosis; cytokinesis; cellular component morphogenesis | PH day 7 |
| **1061** | Actin, alpha skeletal muscle | ACTA1 | intracellular protein transport; exocytosis; endocytosis; mitosis; cytokinesis; cellular component morphogenesis | PH day 7 |
| **1076** | Alpha-2-HS-glycoprotein (Fragment) | AHSG | immune system process; protein metabolic process; mesoderm development; skeletal system development | TC – HDrip |
| **1078** | Enolase | ENO3 | glycolysis | TC – HDrip |
| **1078** | Beta-enolase | ENO3 | glycolysis | TC – HDrip |
| **1078** | Aspartoacylase | ASPA | metabolic process | TC – HDrip |
| **1078** | L-lactate dehydrogenase A chain | LDHA | tricarboxylic acid cycle; glycolysis | TC – HDrip |
| **1078** | Malate dehydrogenase, cytoplasmic | MDH1 | tricarboxylic acid cycle; carbohydrate metabolic process | TC – HDrip |
| **1078** | Aldose reductase | AKR1B1 | metabolic process | TC – HDrip |
| **1078** | L-lactate dehydrogenase B chain | LDHB | tricarboxylic acid cycle; glycolysis | TC – HDrip |
| **1078** | L-lactate dehydrogenase C chain | Q9TSX5 | tricarboxylic acid cycle; glycolysis | TC – HDrip |
| **1078** | Fructose-1,6-bisphosphatase 1 | FBP1 | carbohydrate metabolism; gluconeogenesis | TC – HDrip |
| **1192** | Heat shock 70 kDa protein 1A | HSPA1A | immune system process; protein metabolic process; response to stress | TC - LDrip |
| **1192** | Serum albumin | ALB | transport | TC - LDrip |
| **1192** | Heat shock 70 kDa protein 6 | HSPA6 | immune system process; protein metabolic process; response to stress | TC - LDrip |
| **1192** | Heat shock 70 kDa protein 1B | HSPA1B | immune system process; protein metabolic process; response to stress | TC - LDrip |
| **1192** | Heat shock 70 kDa protein 1-like | HSPA1L | immune system process; protein metabolic process; response to stress | TC - LDrip |
| **1264** | Heat shock 70kDa protein 1A | HSPA1A | immune system process; protein metabolic process; response to stress | TC – HDrip |
| **1264** | Serum albumin | ALB | transport | TC – HDrip |
| **1264** | Heat shock 70 kDa protein 1B | HSPA1B | immune system process; protein metabolic process; response to stress | TC – HDrip |
| **1264** | Heat shock 70 kDa protein 6 | HSPA6 | immune system process; protein metabolic process; response to stress | TC – HDrip |
| **1264** | Heat shock 70 kDa protein 1-like | HSPA1L | immune system process; protein metabolic process; response to stress | TC – HDrip |
| **1279** | Ig lambda chain C region | IGLC1 | antigen binding | TC - LDrip |
| **1287** | Rho GDP-dissociation inhibitor 1 | ARHGDIA | intracellular signaling cascade; signal transduction | TC – HDrip; TC - LDrip |
| **1287** | Guanidinoacetate N-methyltransferase | GAMT | creatine biosynthetic process; muscle contraction; small molecule metabolic process | TC – HDrip; TC - LDrip |
| **1290** | Heat shock 70 kDa protein 1A | HSPA1A | immune system process; protein metabolic process; response to stress | TC - LDrip |
| **1290** | Heat shock 70 kDa protein 6 | HSPA6 | immune system process; protein metabolic process; response to stress | TC - LDrip |
| **1290** | Serum albumin | ALB | transport | TC - LDrip |
| **1290** | Myc box-dependent-interacting protein 1 | BIN1 | [neurotransmitter secretion](http://www.pantherdb.org/panther/category.do?categoryAcc=GO:0007269); [intracellular protein transport](http://www.pantherdb.org/panther/category.do?categoryAcc=GO:0006886); [endocytosis](http://www.pantherdb.org/panther/category.do?categoryAcc=GO:0006897); [synaptic transmission](http://www.pantherdb.org/panther/category.do?categoryAcc=GO:0007268); [cell-cell signaling](http://www.pantherdb.org/panther/category.do?categoryAcc=GO:0007267) | TC - LDrip |
| **1290** | Heat shock 70 kDa protein 1B | HSPA1B | immune system process; protein metabolic process; response to stress | TC - LDrip |
| **1290** | Heat shock 70 kDa protein 1-like | HSPA1L | immune system process; protein metabolic process; response to stress | TC - LDrip |
| **1290** | Glucose-6-phosphate isomerase | GPI | gluconeogenesis; glycolysis | TC - LDrip |
| **1360** | Alpha-2-HS-glycoprotein (Fragment) | AHSG | immune system process; protein metabolic process; mesoderm development; skeletal system development | TC – HDrip |

^a^Spot numbers refer to Fig 3 in [15]. ^b^Biological process of the proteins obtained using PANTHER analysis [25]. ^c^Comparison where the protein was significantly different [phenotype (PH), time course (TC)].
